# Aptamer‐Engineered Ellipsometry for Clinical Detection of BALF‐Derived Exosomes: Multi‐Level Engineering for Prognostic Evaluation of Immunotherapy Responses

**DOI:** 10.1002/advs.202515247

**Published:** 2025-10-31

**Authors:** Euna Jeong, Jung Hyun Choi, Minyoung Lee, Jun Hyeok Lim, Seokho Jung, Yeeun Woo, Joo‐young Kim, Jaehyeon Ok, Lucia Kim, Woo‐jin Jeong, Won Chegal, Hyun Mo Cho, Chulhwan Park, Jiyoon Bu, Dong Hyung Kim, Taek Lee

**Affiliations:** ^1^ Department of Biological Sciences and Bioengineering Inha University Incheon 22212 Republic of Korea; ^2^ Division of Biomedical Metrology Korea Research Institute of Standards and Science Daejeon 34113 Republic of Korea; ^3^ Department of Chemical Engineering Kwangwoon University Seoul 01897 Republic of Korea; ^4^ Division of Pulmonology Department of Internal Medicine Inha University Hospital Inha University College of Medicine Incheon 22332 Republic of Korea; ^5^ Department of Pathology Inha University Hospital Inha University College of Medicine Incheon 22332 Republic of Korea; ^6^ Strategic Technology Research Institute Korea Research Institute of Standards and Science Daejeon 34113 Republic of Korea; ^7^ SIS sensor corporation 267 Gajeongno Yuseong‐Gu Daejeon 34113 Republic of Korea; ^8^ Biohybrid Systems Research Center Inha University Incheon 22212 Republic of Korea; ^9^ Department of Medicinal Biosciences and Bioengineering Inha University Incheon 22212 Republic of Korea; ^10^ Department of Applied Measurement Science University of Science and Technology (UST) 217 Gajeong‐ro Yuseong‐gu Daejeon 34113 Republic of Korea

**Keywords:** aptamer truncation, bronchoalveolar lavage fluid, ellipsometry‐based solution immersed silicon biosensor, immunotherapy response prediction, PD‐L1‐expressing exosome detection

## Abstract

Exosomes emerges as indicators of the tumor microenvironment, yet their predictive utility for immunotherapy responses is limited by the insufficient sensitivity and specificity of currently available assays. Here, a multi‐level engineering strategy is presented that enables accurate exosome‐based prediction of immunotherapy responses by integrating systematic aptamer ligand tailoring, ultrasensitive ellipsometry‐based sensing, and clinically relevant tumor‐proximal fluid sampling. Aptamers specifically targeting PD‐L1 are identified through systematic evolution of ligands by exponential enrichment (SELEX), followed by truncation and computational sequence refinement to enhance binding specificity. The optimized aptamer sequence (Tr‐Apt13) is validated from molecular interaction analyses to in vitro assays, demonstrating superior target binding efficacy over conventional antibodies, attributed to dense surface immobilization and multivalent binding capability. When incorporated into an ellipsometry‐based dual‐prism solution‐immersed silicon sensor, Tr‐Apt13 enabled ultrasensitive detection of PD‐L1–expressing exosomes with a detection limit of ≈9.8 particles mL^−1^, exhibiting high linearity and stability. Clinical validation using bronchoalveolar lavage fluid (BALF) from lung cancer patients revealed precise discrimination between responders and non‐responders at exosome concentrations as low as 10^3^ particles mL^−1^, outperforming serum‐based analysis, conventional ELISA, and tissue immunohistochemistry. Collectively, this multi‐level strategy offers a new perspective on exosome‐based diagnostics, enabling precise prediction of immunotherapy efficacy in lung cancer patients.

## Graphical abstract

A novel ellipsometry platform functionalized with truncated PD‐L1 aptamers are developed to enable ultrasensitive detection of BALF‐derived exosomes. Through chemical ligand tailoring, mechanical sensing optimization, and tumor‐proximal fluid sampling, the system precisely stratifies ICI responders and non‐responders, offering superior sensitivity and clinical applicability over conventional assays, with strong potential to advance personalized immunotherapy.

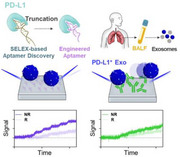

## 1 Introduction

Immune checkpoint inhibitors (ICIs), particularly those targeting programmed death 1 (PD‐1) and its counter ligand PD‐L1, have demonstrated substantial therapeutic efficacy across various malignancies by restoring T cell–mediated antitumor immunity.^[^ ^1^, ^2^ ^]^ Despite their clinical success, response rates remain limited, with objective responses observed in only 7–13% of tumors exhibiting low PD‐L1 expression.^[^ ^3^, ^4^, ^5^, ^6^ ^]^ Currently, PD‐L1 immunohistochemistry (IHC) serves as the standard companion diagnostic; however, its predictive accuracy is limited by spatial and temporal heterogeneity, as well as the invasive nature of tissue sampling. Exosomal PD‐L1 has emerged as an alternative biomarker that more accurately reflects the dynamic immunological state of tumors.^[^ ^7^, ^8^ ^]^ Exosomes, extracellular vesicles released by nearly all cell types, are abundant in bodily fluids and can carry membrane‐bound proteins such as PD‐L1, particularly when originating from tumor cells.^[^ ^9^, ^10^ ^]^ ELISA‐based analyses have shown that plasma exosomal PD‐L1 levels can exceed 100 pg mL^−1^ in patients with non‐small cell lung cancer (NSCLC), with higher levels associated with poor ICI response and shorter progression‐free survival (PFS).^[^ ^11^ ^]^ Several clinical studies have reported that baseline exosomal PD‐L1 more effectively differentiates responders from non‐responders than soluble PD‐L1 or tumor PD‐L1 assessed by IHC, suggesting its potential as a sensitive biomarker for predicting immunotherapy outcomes.^[^ ^11^, ^12^ ^]^

Although ELISA remains the gold standard for quantifying exosomal PD‐L1 levels, it is limited by low sensitivity, a narrow dynamic range, and operator‐dependent variability.^[^ ^13^, ^14^ ^]^ Moreover, the heterogeneity of circulating exosomes, which originate from both tumor and non‐tumor sources, compromises the specificity of ELISA‐based detection for tumor‐derived PD‐L1.^[^ ^15^, ^16^ ^]^ To address these limitations, a variety of biosensing technologies have been developed to enhance analytical performance. Optical biosensors such as surface plasmon resonance (SPR) and surface‐enhanced Raman spectroscopy (SERS) enable real‐time, label‐free detection of exosomal surface markers. For instance, localized SPR using core–shell nanoparticles has achieved detection limits as low as 1.2 × 10⁶ particles mL^−1^, while SERS‐based platforms provide broad dynamic detection ranges.^[^ ^17^, ^18^ ^]^

Most existing exosome detection biosensors utilize antibodies as capture agents due to their high affinity and established specificity. However, antibody‐based systems present several intrinsic limitations, including high production costs, batch‐to‐batch variability, and structural instability under non‐physiological conditions.^[^ ^19^, ^20^ ^]^ As an alternative, aptamers—short, single‐stranded oligonucleotides typically selected via Systematic Evolution of Ligands by Exponential Enrichment (SELEX)— offer synthetic accessibility, chemical stability, and cost‐effective production, along with precise and reproducible surface functionalization.^[^ ^21^, ^22^ ^]^ Although aptamers typically exhibit lower binding affinity than antibodies in solution, their integration into biosensor platforms enables comparable detection sensitivity.^[^ ^23^, ^24^ ^]^ Moreover, aptamers can be further engineered to enhance their performance through strategies such as sequence optimization, structural modification, and chemical conjugation. For instance, truncation of non‐essential regions can yield shorter constructs with retained or improved affinity and reduced synthesis costs.^[^ ^25^, ^26^ ^]^ These advantages collectively position aptamers as effective and versatile recognition elements for the selective capture of diverse biomolecules in various biosensing platforms.

In this study, we hypothesized that aptamers specifically engineered to recognize PD‐L1 could serve as effective capture agents for detecting low‐abundance PD‐L1–expressing exosomes when integrated into an ultrahigh‐sensitivity optical sensing platform. To maximize sensitivity and specificity, we applied three complementary engineering strategies addressing chemical, mechanical, and clinical aspects: i) a chemical strategy involving truncation of SELEX‐derived PD‐L1–targeting aptamers to simplify the sequence while preserving core binding motifs. This approach addresses the presence of redundant regions in SELEX‐derived aptamers (e.g., primer‐binding sites) that may compromise structural stability and binding specificity under physiological conditions; ii) a mechanical strategy applying these truncated aptamers to a dual‐prism solution‐immersed silicon (DP‐SIS) ellipsometry sensor, originally developed for real‐time thin‐film monitoring, which was adapted for ultrahigh‐sensitivity, label‐free detection of exosomal surface markers through selective molecular recognition^[^ ^27^, ^28^ ^]^; and iii) a clinical strategy utilizing bronchoalveolar lavage fluid (BALF) instead of serum as the sample source, as BALF can be collected directly from tumor proximal sites, potentially enhancing detection specificity for lung cancer–associated exosomes.

To test these hypotheses, two PD‐L1–specific aptamer sequences (Apt13 and Apt45) were selected via SELEX. Through computational analyses, combined with molecular binding assays and in vitro affinity measurements, we identified an optimized sequence and further engineered it through truncation to enhance target binding and specificity (Tr‐Apt13). Multiple validation methods, including molecular docking simulations, molecular interaction measurements, and cell retention assay, confirmed that Tr‐Apt13 exhibited higher in vitro targeting efficiency than its antibody counterpart (aPD‐L1), attributed to multivalent binding effects. This enhanced in vitro targeting efficiency translated into improved sensitivity and specificity for PD‐L1–expressing exosome capture when applied to the ultrahigh‐sensitivity DP‐SIS sensor, significantly outperforming both aPD‐L1–functionalized sensors and conventional ELISA. Using patient‐derived BALF samples, we further demonstrated the clinical utility of our system by comparing the predictive performance of PD‐L1–positive exosomes with that of conventional tissue PD‐L1 IHC staining, exosomal PD‐L1 ELISA, and aPD‐L1–functionalized sensors. Additionally, we confirmed that BALF‐based analysis provided improved predictive performance compared to serum‐based approaches, owing to its inherent proximity to tumor sites and higher enrichment of tumor‐derived exosomes. These findings establish the aptamer‐engineered DP‐SIS sensor as a clinically applicable platform for sensitive detection of BALF‐derived PD‐L1‐expressing exosomes, improving prediction of ICI response in lung cancer.

## 2 Results

### 2.1 Overall Design of the Aptamer‐Engineered DP‐SIS Sensor

**Figure 1:**
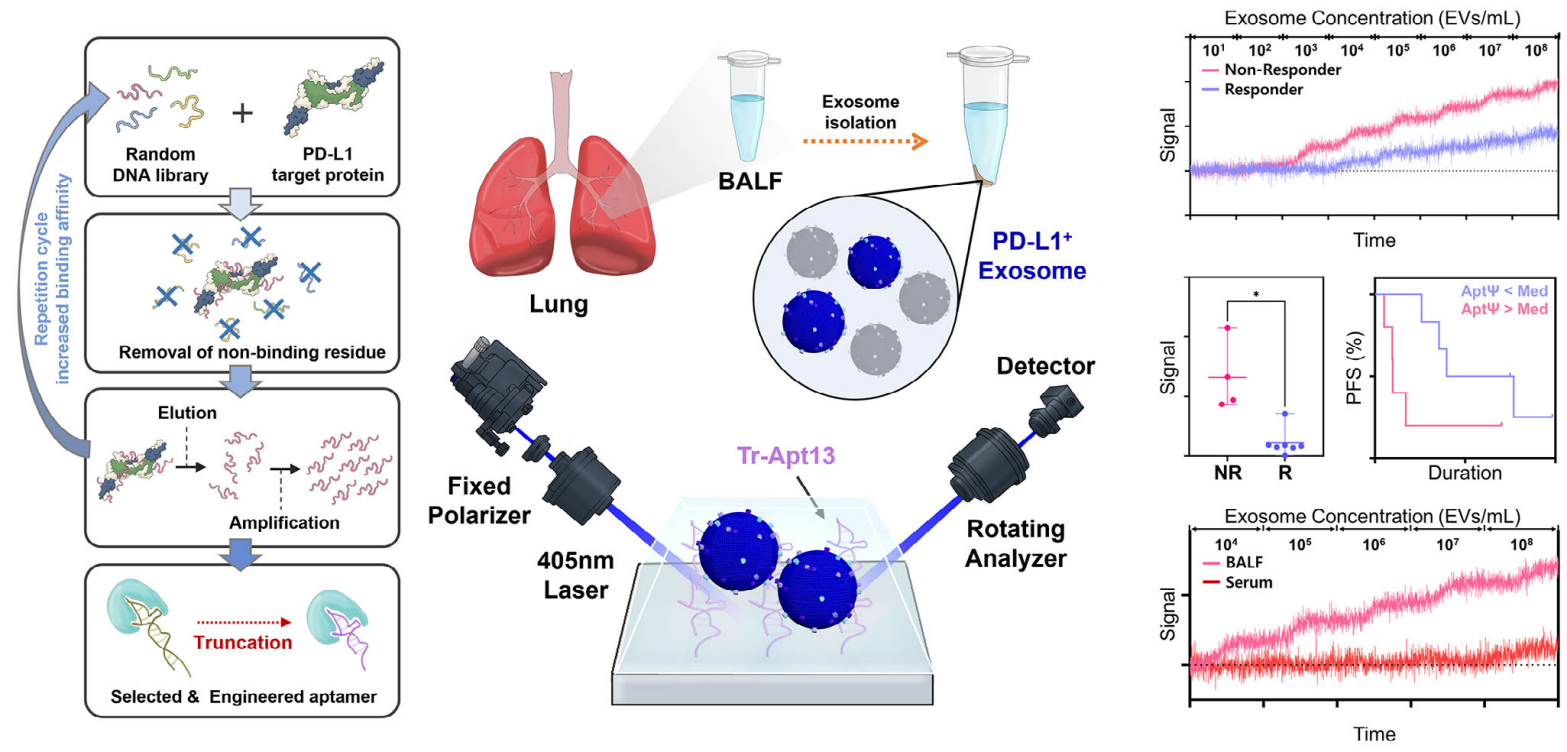
Schematic overview of the aptamer‐engineered DP‐SIS sensor platform for ultrasensitive detection of PD‐L1–expressing exosomes from BALF. The SELEX process was used to isolate PD‐L1–specific aptamers from a random DNA library, and the optimized sequence was truncated to enhance target specificity (left). The truncated aptamer (Tr‐Apt13) was then immobilized on the DP‐SIS sensor for exosome detection (center). For clinical validation, PD‐L1–expressing exosomes in BALF were analyzed using the Tr‐Apt13–immobilized DP‐SIS sensor to predict ICI treatment responses, and the results were compared with those obtained from serum samples (right).

The overall design of the aptamer‐engineered DP‐SIS sensor platform for detecting PD‐L1–expressing exosomes from BALF is illustrated in **Figure** **1**. Aptamer sequences with high binding specificity toward recombinant PD‐L1 were obtained through ten rounds of an iterative SELEX process. Among the identified candidates, two aptamer sequences (Apt13 and Apt45) were further evaluated and optimized through multiple binding interaction assays. Apt13, which exhibited superior target specificity and binding affinity, was subsequently truncated to enhance binding performance and reduce molecular size, yielding the final sequence Tr‐Apt13.

Tr‐Apt13 was then immobilized onto the DP‐SIS sensor and applied for the detection of PD‐L1–expressing exosomes from BALF. The DP‐SIS sensor setup consisted of a diode laser (center wavelength: 405 nm, output power: 900 µW), a rotating polarizer, a sensor cell assembly, an analyzer, and silicon photodiode detectors. The linearly polarized laser beam was directed onto the sensor surface, and the reflected elliptically polarized light was analyzed by the rotating polarizer, separating it into its *p*‐ and *s*‐polarized components to monitor changes in the overlayer thickness induced by biomolecular interactions on the sensor surface. The real‐time changes in the amplitude ratio (Ψ) of these components were recorded, corresponding to the number of exosomes captured on the sensor surface. Upon confirmation of performance with in vitro samples, the Tr‐Apt13–immobilized DP‐SIS sensor was applied to BALF samples from lung cancer patients undergoing ICI therapy to evaluate its ability to discriminate between responders and non‐responders.

### 2.2 Systematic Selection, Optimization, and Validation of PD‐L1–Targeting Aptamers

Ten rounds of an iterative SELEX process were conducted to obtain aptamer sequences exhibiting high specificity toward recombinant PD‐L1. The DNA library used in the SELEX process was designed with fixed primer binding sites at both ends and a central region of 40 randomized nucleotides. While this library configuration theoretically enables a sequence diversity of ≥ 4^30^ (≈1.2 × 10^18^), practical constraints in oligonucleotide synthesis and handling limit the number of accessible sequences to ≈6 × 10^13^–6 × 10^14^.^[^ ^29^, ^30^ ^]^ Even within these limitations, the 40‐nucleotide random region provides sufficient structural complexity to support the formation of diverse secondary and tertiary structures, while minimizing nonspecific interactions and sequence redundancy. As a result, random regions of 30–50 nucleotides have been widely adopted as an optimal design for SELEX‐based aptamer selection. Previous studies have demonstrated that such a library design can successfully yield high‐affinity aptamers targeting flavivirus‐derived proteins.^[^ ^31^ ^]^ Based on these findings, the present study applied the same SELEX conditions, under which binding specificity and structural stability improved progressively throughout the selection process. Confirmation was provided by Tris‐Borate‐EDTA polyacrylamide gel electrophoresis (TBE‐PAGE) analysis of the initial and final SELEX products (Figure S1, Supporting Information). Compared with control proteins such as bovine serum albumin (BSA) and myoglobin, the selected aptamers exhibited high specificity toward PD‐L1, indicating that target binding had reached saturation after ten rounds of selection without the need for additional cycles.

**Figure 2:**
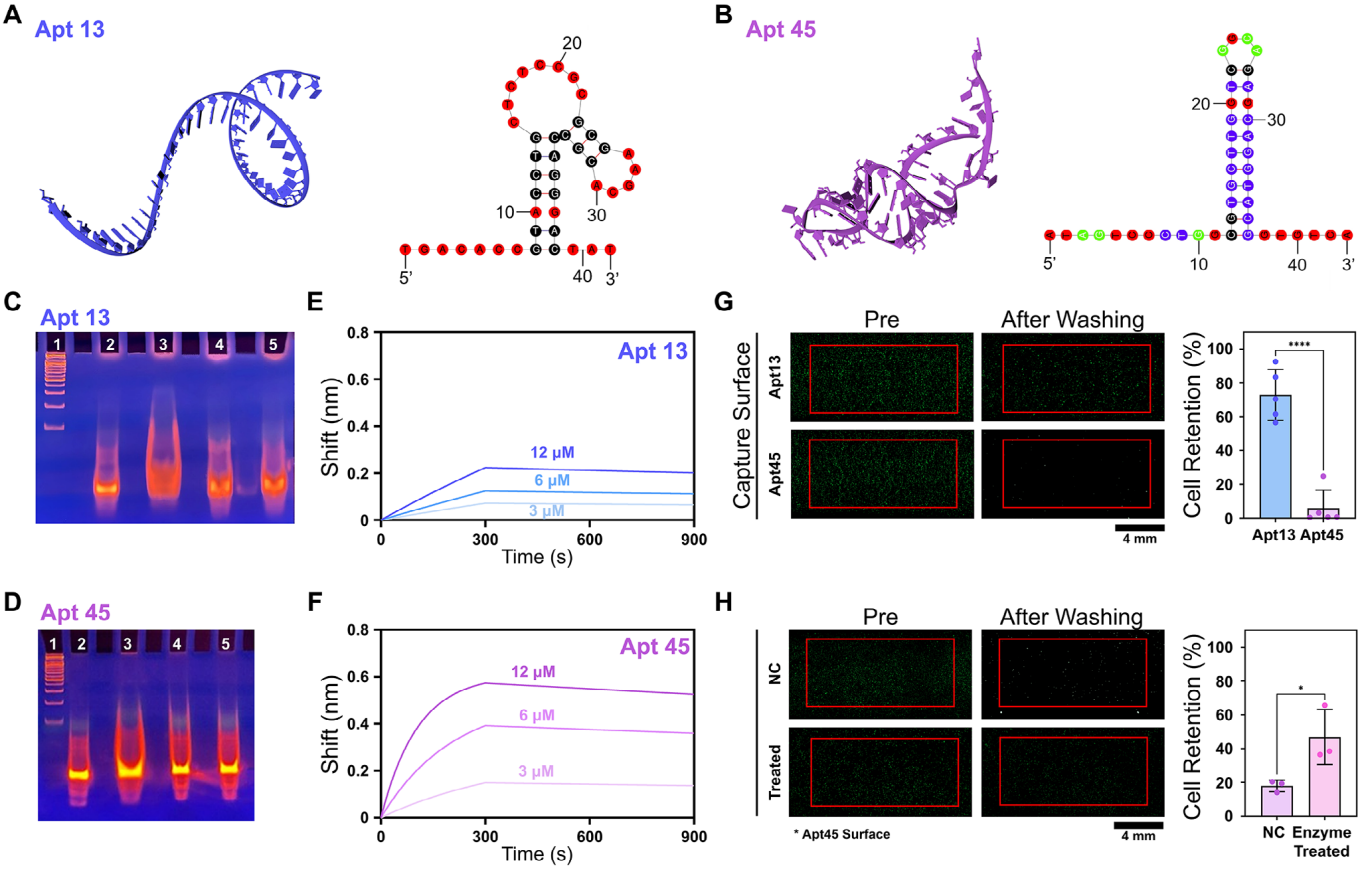
Selection, structural characterization, and functional validation of PD‐L1–targeting aptamers. A,B) Predicted 3D and 2D structures of Apt13 and Apt45 with calculated Gibbs free energy values, indicating canonical stem‐loop conformations with high thermodynamic stability. In the 2D structures, colors represent base‐pairing status and functional roles: Red, unpaired bases (loop regions, potential target‐interaction sites); Black, stem regions (double‐helix stabilization); Purple, thermodynamically stable G–C base pairs; Green, functionally important unpaired bases (structural flexibility and steric interaction potential). Numbers indicate nucleotide positions, and 5′/3′ labels denote sequence directionality. C,D) TBE‐PAGE analysis showing specific interaction of Apt13 and Apt45 with recombinant PD‐L1. Distinct mobility shifts were observed in lanes containing aptamer–PD‐L1 complexes compared to controls. Lane assignments: 1, 100 bp DNA ladder; 2, aptamer; 3, recombinant PD‐L1 + aptamer; 4, BSA + aptamer; 5, myoglobin + aptamer. E,F) BLI analysis of Apt13 and Apt45 binding kinetics toward immobilized PD‐L1, confirming strong molecular‐level affinity. G) Cell retention assay comparing in vitro targeting efficiency of Apt13 and Apt45 using PD‐L1^High^ MDA‐MB‐231 cells, demonstrating significantly higher cell retention for Apt13. H) Cell retention assay following enzymatic deglycosylation of MDA‐MB‐231 cells with PNGase F, confirming that the low in vitro targeting efficiency of Apt45 is attributable to glycosylation of membrane‐bound PD‐L1. Note that the significance levels are indicated as ^*^ *p* < 0.05 and ^****^ *p* < 0.0001, determined by two‐sided Student's *t*‐tests (*n* ≥ 3).

The final SELEX product was subsequently refined into 50 unique sequences through sequencing analysis. Predicted 2D and 3D structures of these aptamer candidates were modeled using RNA Composer, and their thermodynamic parameters, including Gibbs free energy (Δ*G*), were calculated using UNAFold. Among these, Apt13 and Apt45, which adopt canonical stem‐loop structures (**Figure** **2**A,B) and exhibited the highest thermodynamic stability with Δ*G* of −6.16  and −7.16 kcal mol^−1^, respectively, were selected as the final candidates (Table S1, Supporting Information). Considering that the stem region contributes structural rigidity while the loop region serves as the primary site for target recognition, these predicted conformations indicate strong functional suitability for PD‐L1 binding (See the Note S1, Supporting Information for further details). In particular, the formation of a unique 3D structure facilitates a stable binding network through non‐covalent interactions such as hydrogen bonding and *π*–*π* stacking with specific amino acid residues in the target protein, including Tyr56, Tyr123, and Arg113 within the IgV domain.^[^ ^32^ ^]^ The guanine‐rich sequence located in the loop region plays a critical role in mediating these interactions, thereby enhancing both binding affinity and specificity.^[^ ^33^, ^34^ ^]^ TBE‐PAGE analysis confirmed that both Apt13 and Apt45 maintained robust binding affinity for PD‐L1, with negligible interference from overlapping sequences or steric hindrance (Figure 2C,D).

Binding kinetics of the selected aptamers were further evaluated using biolayer interferometry (BLI). Recombinant PD‐L1 was immobilized on the sensor surface, and the real‐time binding interaction of the aptamers in aqueous solution was monitored (Figure 2E,F). Both Apt13 and Apt45 exhibited strong binding to recombinant PD‐L1, with dissociation constants (K_D_) of ≈7.14 × 10^2 ^nm and ≈1.86 × 10^2^ nm, respectively. Notably, these values indicate significantly higher affinity than that of the native PD‐1–PD‐L1 interaction, which has been reported in previous studies to fall within the micromolar range.^[^ ^35^, ^36^ ^]^

Prior to applying these aptamers for exosome capture, we evaluated their target‐binding efficiency using a cell retention assay, as binding behavior can differ between soluble protein assays and cell‐ or exosome‐associated targets due to the conformational and structural context of membrane‐anchored proteins.^[^ ^37^, ^38^, ^39^ ^]^ A flow chamber equipped with glycine‐coated basal slides functionalized with either Apt13 or Apt45 was utilized for the assay (Figure S2, Supporting Information). In brief, PD‐L1^High^ MDA‐MB‐231 cells were incubated in the chamber for 10 min, followed by sequential washing under increasing shear flow rates (50–1000 µL min^−1^) to assess in vitro targeting efficiency based on cell retention (Figure S3, Supporting Information). Although Apt45 exhibited stronger binding kinetics at the molecular level, the cell retention assay showed negligible cell adhesion on the Apt45‐functionalized surface (retention rate: 5.9 ± 10.0%), whereas Apt13 showed a significantly higher cell retention rate of 72.9 ± 13.5% (*p* < 0.0001) (Figure 2G).

We hypothesized that the discrepancy between molecular‐level binding affinity and in vitro cell‐binding efficiency is attributed to the structural features of membrane‐bound PD‐L1. Glycosylation, a well‐established post‐translational modification of PD‐L1, has been shown to hinder ligand recognition and reduce binding affinity, particularly through N‐glycans present on its plasma membrane–localized form.^[^ ^40^, ^41^ ^]^ To test this hypothesis, cells were pre‐treated with PNGase F to enzymatically remove N‐linked glycans.^[^ ^40^ ^]^ The deglycosylated MDA‐MB‐231 cells exhibited significantly enhanced cell retention (46.0 ± 13.3%; *p* = 0.040), confirming that glycosylation impairs Apt45 binding on the cell surface (Figure 2H). Note that the 3 h PNGase F treatment did not affect cell viability (Figure S4, Supporting Information) or adhesion of PD‐L1–negative control cells (Figure S5, Supporting Information). Despite this, Apt13 demonstrated consistently higher in vitro targeting efficiency compared to Apt45, indicating stronger binding to membrane‐bound PD‐L1. Taken together, Apt13 was selected as the capture agent for subsequent truncation engineering and exosome capture.

### 2.3 Truncation and Validation of Optimized Sequence for PD‐L1^+^ Biomarker Detection

**Figure 3:**
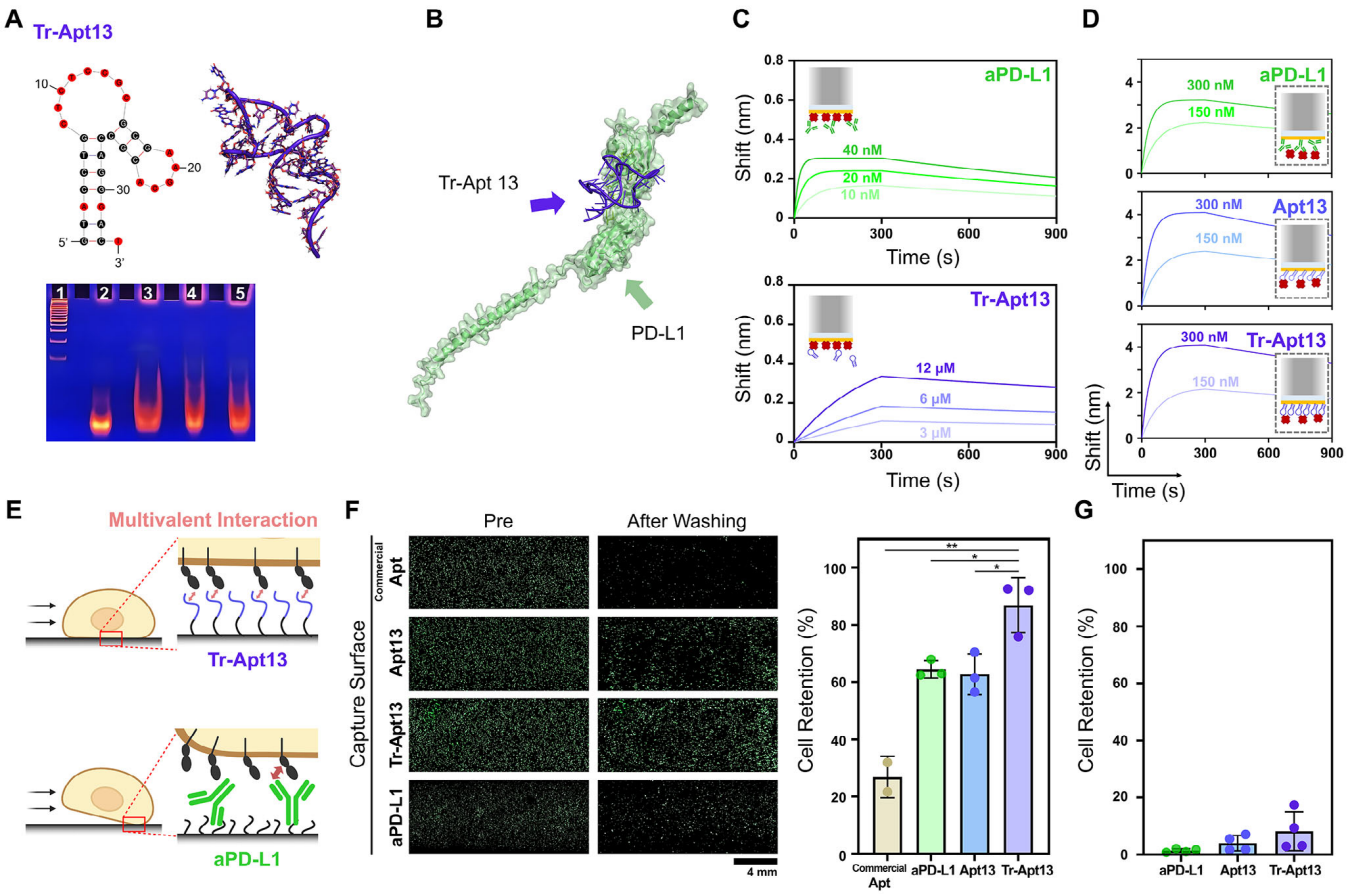
Truncation engineering and functional validation of Tr‐Apt13 for PD‐L1⁺ biomarker detection. A) Predicted 2D and 3D structures of Tr‐Apt13 and TBE‐PAGE analysis confirming its specific binding to PD‐L1 with no binding to control proteins. Lane assignments: 1, 100 bp DNA ladder; 2, Tr‐Apt13; 3, recombinant PD‐L1 + Tr‐Apt13; 4, BSA + Tr‐Apt13; 5, myoglobin + Tr‐Apt13. B) Molecular docking simulation of the PD‐L1–Tr‐Apt13 complex, indicating high binding affinity and predictive reliability. C) BLI sensorgrams showing binding kinetics of Tr‐Apt13 and aPD‐L1 toward PD‐L1. D) Inverse BLI assay comparing binding responses of aptamer‐ and antibody‐functionalized sensor surfaces. E) Schematic illustration of multivalent binding interactions achieved by Tr‐Apt13 versus aPD‐L1 due to higher surface density and favorable orientation. F) Cell retention assay using PD‐L1^High^ MDA‐MB‐231 cells, showing significantly higher cell adhesion on Tr‐Apt13–functionalized surfaces compared to Apt13, aPD‐L1, and a commercial PD‐L1 aptamer. G) Specificity assessment using PD‐L1^Negative^ HL‐60 cells, confirming minimal nonspecific retention on Tr‐Apt13–functionalized surfaces. Note that the significance levels are indicated as ^*^ *p* < 0.05 and ^**^ *p* < 0.01, determined by two‐sided Student's *t*‐tests (*n* ≥ 3).

SELEX‐derived aptamers typically possess relatively long sequences comprising fixed primer‐binding regions flanking a central randomized sequence, which may result in the inclusion of overlapping sequences such as primer‐binding sites. These overlapping regions can adversely affect binding specificity and facilitate nonspecific interactions.^[^ ^26^, ^34^, ^42^, ^43^, ^44^, ^45^ ^]^ Previous studies have further demonstrated that aptamer binding affinity and specificity are often restricted to well‐defined structural motifs, such as stem‐loop regions.^[^ ^46^ ^]^ To address this limitation, we truncated Apt13 to generate an optimized variant, Tr‐Apt13 (Table S1, Supporting Information). Note that the retained sequence regions after truncation are highlighted with a blue background (Figure S6, Supporting Information). A secondary structure prediction tool was utilized to perform a detailed analysis of the structural characteristics of the full‐length aptamer. Based on these findings, non‐functional or structurally independent terminal regions were systematically removed. The ΔG of Tr‐Apt13 was determined as −5.73 kcal mol^−1^, slightly higher than that of the original Apt13 (−6.16 kcal mol^−1^). This minor difference arises from the methodological aspect of Δ*G* estimation, which involves the summation of the free energy contributions from both the stem and loop structures. Nevertheless, the predicted 2D and 3D models showed that the overall stem‐loop structure was not substantially altered despite the truncation, and the loop region essential for target recognition was preserved. Consistently, no adverse effect on binding performance was observed in subsequent assays. TBE‐PAGE analysis confirmed that Tr‐Apt13 maintained distinct band shifts in the presence of PD‐L1, whereas no such shift was observed with control proteins, further validating its PD‐L1 binding specificity. These findings indicate that structural simplification of Apt13 preserved functional integrity, supporting stable aptamer–target complex formation (**Figure** **3**A).

To further delineate the binding interface of Tr‐Apt13, molecular docking simulations were performed (Figure 3B). Site‐directed mutational analyses focused on nucleotides within the stem and loop regions were incorporated to assess binding contributions. The binding accuracy of each model was evaluated using docking scores, ligand RMSD values, and confidence scores. Typically, a lower docking score reflects stronger binding energy, a lower ligand RMSD indicates precise binding site localization, and a confidence score exceeding 0.7 denotes a reliable binding conformation.^[^ ^47^, ^48^ ^]^ Docking simulations revealed a docking score of −281.52, ligand RMSD of 63.20 Å, and a confidence score of 0.9328 for the PD‐L1–Tr‐Apt13 complex, indicating high binding affinity and predictive reliability (Table S2, Supporting Information).

To experimentally validate the binding performance of Tr‐Apt13 following truncation and in silico modeling, BLI analysis was conducted (Figure 3C). Tr‐Apt13 exhibited a K_D_ of ≈9.91 × 10^2 ^nm, comparable to that of its original form, Apt13 (≈7.14 × 10^2 ^nm), indicating that truncation did not compromise its binding affinity toward PD‐L1. However, both aptamers showed significantly higher dissociation constants compared to their antibody counterpart (aPD‐L1), which exhibited a K_D_ of ≈3.86 × 10^2^ pM.

Although aptamers generally exhibit lower binding affinity than antibodies in solution‐phase comparisons, their performance as capture agents can differ when immobilized onto a sensing surface.^[^ ^49^ ^]^ Due to their smaller molecular size and well‐defined orientation upon surface immobilization, aptamers allow for higher surface density and more consistent exposure of their binding domains.^[^ ^16^ ^]^ To investigate this, we performed an inverse BLI assay by immobilizing either aptamers or antibodies on the sensor chip and measuring the binding response to recombinant PD‐L1 in solution (Figure 3D). In this setup, both Tr‐Apt13 and Apt13 exhibited even higher overall signal intensity than the antibody, supporting their effective surface immobilization for capturing specific biomolecules.

When targeting biomolecules that present multiple surface proteins (i.e., cells or exosomes), this substantially higher surface density and consistent orientation of aptamers becomes particularly advantageous, as it facilitates multivalent binding interactions with target molecules^[^ ^16^, ^50^ ^]^ (Figure 3E). To validate this, a cell retention assay was performed, as described previously. The Tr‐Apt13–immobilized surface exhibited significantly stronger adhesion to PD‐L1^High^ MDA‐MB‐231 cells compared to aPD‐L1–immobilized surface (86.8 ± 7.8% vs 64.4 ± 2.5%; *p* = 0.045), supporting enhanced capture of PD‐L1–expressing cells through multivalent binding to membrane‐bound PD‐L1s (Figure 3F). Notably, Tr‐Apt13 also demonstrated a higher cell retention rate than both Apt13 (62.8 ± 5.8%; *p* = 0.028) and a commercially available PD‐L1–targeting aptamer (26.8 ± 5.2%; *p* = 0.005),^[^ ^51^ ^]^ which may be attributed to the formation of a more stable tertiary structure, which reduces steric hindrance upon immobilization on the sensor surface and enhances the spatial accessibility of the target‐binding domain.^[^ ^52^, ^53^ ^]^ Tr‐Apt13 also maintained high target specificity (Figure 3G), as confirmed by the minimal retention rate observed with PD‐L1^Negative^ HL‐60 cells (8.2 ± 5.9%) under equivalent flow conditions (Figures S7 and S8, Supporting Information). These findings demonstrate that Tr‐Apt13 has the potential to be effectively utilized as a capture agent for PD‐L1–expressing exosome‐sensing platforms, owing to its high binding specificity, enhanced surface immobilization efficiency, and superior multivalent binding capability toward membrane‐bound PD‐L1.

### 2.4 Detection of PD‐L1^+^ Exosomes Using Aptamer‐Engineered DP‐SIS Sensor

**Figure 4:**
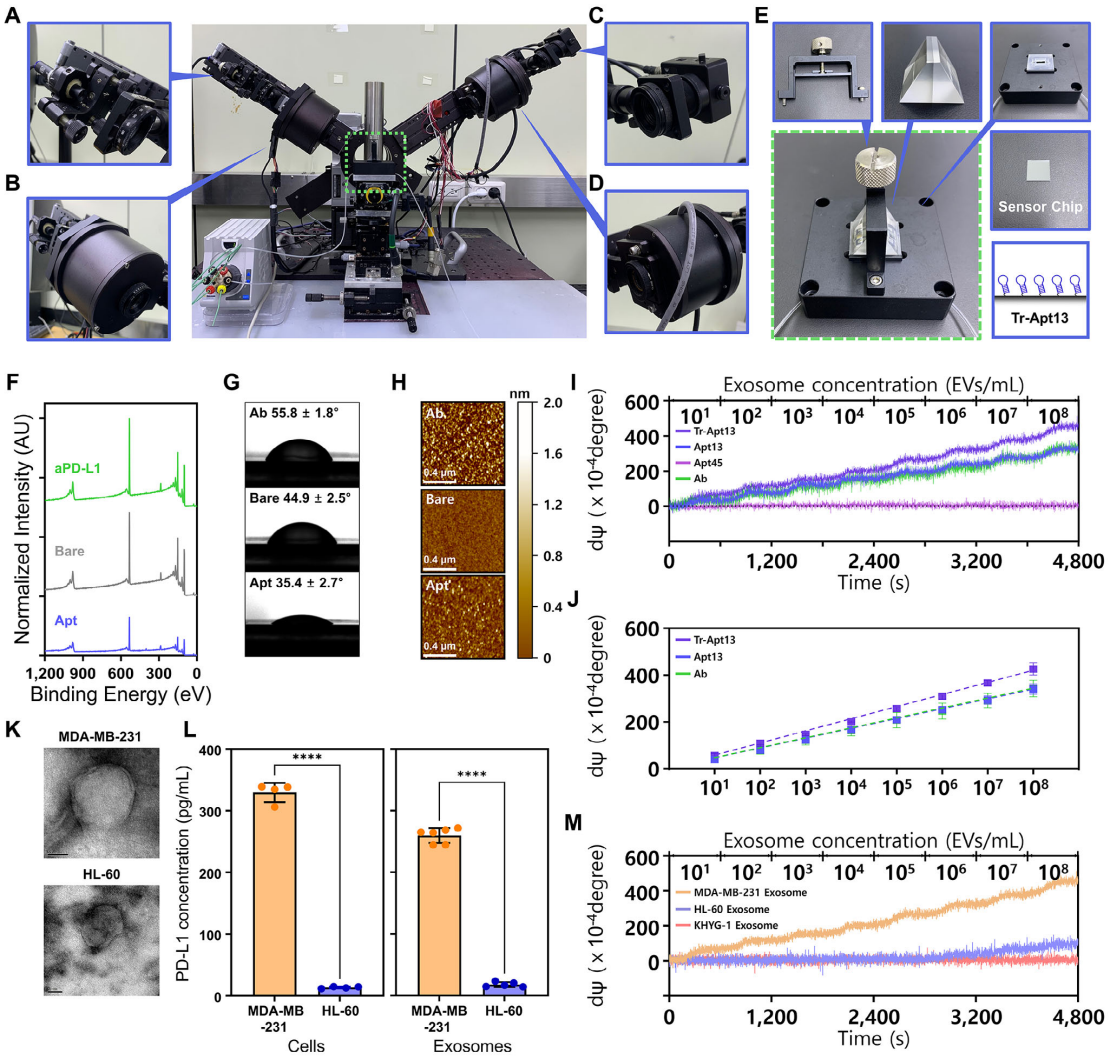
Detection of PD‐L1⁺ exosomes using the Tr‐Apt13–functionalized DP‐SIS sensor. A) Image of DP‐SIS sensor; The sensor consists of (A) a laser diode, B) fixed polarizer, C) detector, D) rotating analyzer, E) DP‐SIS assembly. F) XPS spectra showing characteristic shifts in surface elemental composition upon aptamer or antibody conjugation. G) Sessile drop analysis showing changes in contact angle following aptamer or antibody immobilization. H) AFM images demonstrating increased surface roughness after aptamer or antibody immobilization. I) Real‐time sensograms of polarization shift (dΨ) from DP‐SIS sensor functionalized with Tr‐Apt13, Apt13, Apt45, or aPD‐L1, following the introduction of MDA‐MB‐231–derived exosomes at concentrations from 10^1^ to 10⁸ particles mL^−1^. J) Logarithmic correlation between dΨ and exosome concentration for each sensor surface, with Tr‐Apt13 showing the steepest slope and lowest limit of detection. K) TEM images of MDA‐MB‐231– and HL‐60–derived exosomes confirming similar cup‐shaped morphology (scale bars: 50 nm). L) ELISA quantification of PD‐L1 protein levels in MDA‐MB‐231 and HL‐60 cells and their corresponding exosomes, showing negligible expression in HL‐60 samples. M) Sensogram showing selective detection of MDA‐MB‐231 exosomes by the Tr‐Apt13–DP‐SIS sensor, with no response observed for exosomes derived from PD‐L1^Negative^ cells. Note that the significance levels are indicated as ^****^ *p* < 0.0001, determined by two‐sided Student's *t*‐tests (*n* ≥ 3).

To enable PD‐L1⁺ exosome detection, aptamers were immobilized onto the SiO_2_ surface of the DP‐SIS sensor chip via amine coupling. For comparison, control sensor chips were prepared by immobilizing aPD‐L1 on the sensor surface using the same chemistry. The overall design and structural components of the DP‐SIS sensor system are illustrated in **Figure** **4**A–E. The conjugation of capture binders onto the SiO_2_ surface was confirmed by multiple surface characterization methods. X‐ray photoelectron spectroscopy (XPS) revealed an increase in N1s and C1s signals upon aptamer or antibody conjugation (Figure 4F; Figure S9, Supporting Information). Sessile drop analysis revealed a significant decrease in contact angle after aptamer immobilization, while aPD‐L1 immobilization caused an increase (Figure 4G; Figure S10, Supporting Information). The enhanced hydrophilicity of the aptamer‐modified surface is expected to reduce nonspecific adsorption, potentially improving the specificity of biomolecular interactions while minimizing background noise.^[^ ^54^, ^55^ ^]^ Atomic force microscopy (AFM) further demonstrated an increase in surface roughness after immobilization (Figure 4H), with antibody‐functionalized surfaces exhibiting greater roughness (500 ± 58 pm) than aptamer‐functionalized surfaces (298 ± 12 pm). This implies that aptamers form a more uniform immobilized layer, likely due to their smaller molecular size and the ability to achieve higher surface density with consistent orientation. In contrast, antibodies possess multiple amine groups that promote random immobilization via amine coupling, which may lead to greater surface irregularity, steric hindrance, and reduced efficiency of biomolecular interactions. Ellipsometric signal measurement using DP‐SIS after immobilization of Tr‐Apt13 or aPD‐L1 further supported this finding, as Tr‐Apt13 produced a comparable increase in dΨ to aPD‐L1 (Figure S11, Supporting Information). Given the markedly lower molecular weight of Tr‐Apt13 (≈10.6 kDa) compared to aPD‐L1 (≈150 kDa), this comparable signal suggests that Tr‐Apt13 was more densely immobilized on the sensor surface.

Aptamer‐ or antibody‐immobilized sensor chips were then mounted onto the DP‐SIS sensor, and exosomes derived from PD‐L1^High^ MDA‐MB‐231 cells were introduced at concentrations ranging from 10^1^ to 10^8^ particles mL^−1^. Exosome suspensions were flowed through the sensor at 100 µL min^−1^ for 10 min at each concentration, enabling quantitative evaluation of PD‐L1⁺ exosome capture efficiency via real‐time monitoring of polarization parameter changes. Consistent with the in vitro cell retention assay, the Tr‐Apt13–immobilized surface exhibited significantly higher detection sensitivity for PD‐L1⁺ exosomes compared to surfaces functionalized with Apt13, Apt45, or aPD‐L1. These results suggest that densely immobilized Tr‐Apt13 facilitates multivalent interactions with PD‐L1 on exosomal membranes, thereby outperforming antibody‐based surfaces in detecting exosomes (Figure 4I). Specifically, the limit of detection (LOD) of Tr‐Apt13‐immobilized DP‐SIS sensor was measured to be ≈9.8 × 10^0^ particles mL^−1^, exceeding that obtained from both aPD‐L1 (≈8.0 × 10^1^ particles mL^−1^) and Apt13 (≈3.0 × 10^1^ particles mL^−1^). Note that LOD was determined as the lowest concentration producing a signal exceeding the baseline mean by three standard deviations. The Tr‐Apt13 also showed strong and stable logarithmic correlation with exosome concentrations with a slope of 5.2 × 10^−3^ (R^2^ = 0.991 and SD = 10.41 × 10^−4^ dΨ), also outperforming aPD‐L1 and Apt13 which had slope of 4.3 × 10^−3^ (R^2^ = 0.989 and SD = 26.1 × 10^−4^ dΨ) and 4.3 × 10^−3^ (R^2^ = 0.950 and SD = 11.6 × 10^−4^ dΨ), respectively (Figure 4J). It should also be noted that Apt45, despite exhibiting high binding affinity to soluble PD‐L1, showed no detectable signal within the tested exosome concentration range, consistent with its low targeting efficiency toward membrane‐bound PD‐L1 observed by cell retention assay. In addition, the Tr‐Apt13–modified surface showed greater signal stability, with reduced fluctuations (± 0.0012 dΨ) relative to antibody‐functionalized surfaces (± 0.0017 dΨ), highlighting the advantage of its uniform aptamer immobilization for exosome detection with the DP‐SIS system (Figure S12, Supporting Information). Furthermore, post‐injection of exosome‐specific antibodies (aCD9 and aCD81) after capture of MDA‐MB‐231–derived exosomes confirmed that the captured entities were exosomes, as reflected by an increase in dΨ, whereas IgG produced no significant signal change (Figure S13, Supporting Information).

Target specificity of the Tr‐Apt13–engineered DP‐SIS sensor was further validated using exosomes derived from PD‐L1^negative^ HL‐60 cells. Transmission electron microscopy (TEM) and nanoparticle tracking analysis (NTA) confirmed that HL‐60–derived exosomes exhibited the characteristic cup‐shaped morphology, with diameters ranging from 50 to 200 nm, comparable to exosomes derived from MDA‐MB‐231 cells (Figure 4K; Figure S14, Supporting Information). However, in contrast to MDA‐MB‐231–derived exosomes, ELISA analysis showed that HL‐60–derived exosomes contained negligible levels of PD‐L1, consistent with the PD‐L1 expression profile of their donor cells (Figure 4L). When applied to the Tr‐Apt13‐immobilized DP‐SIS sensor, HL‐60‐derived exosomes generated no detectable signal within exosome concentrations of ≈10^7^ particles mL^−1^ on Tr‐Apt13‐functionalized surface (Figure 4M). Similarly, exosomes derived from PD‐L1^Negative^ human natural killer KHYG‐1 cells also produced no detectable signals, confirming the high target specificity of the platform.

### 2.5 Clinical Relevance of PD‐L1^+^ Exosomes from BALF for the Prediction of ICI Efficacy

**Figure 5:**
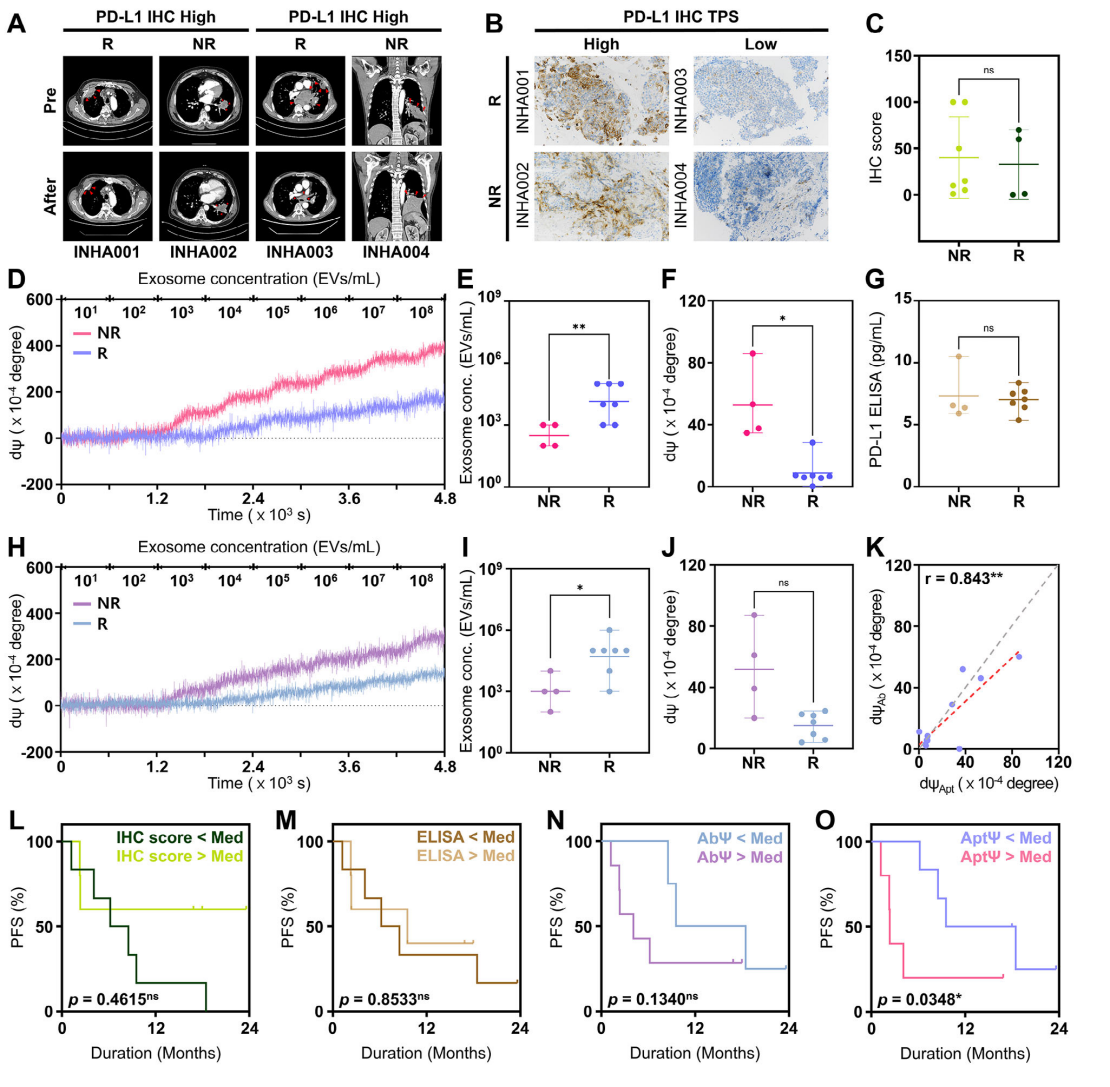
Clinical relevance of Tr‐Apt13–functionalized DP‐SIS sensor for predicting ICI responses using BALF‐derived PD‐L1⁺ exosomes. A) CT images of representative responders and non‐responders before and after ICI treatment. B) IHC images showing PD‐L1 expression in tumor tissues. C) Low predictive capability of tissue TPS for predicting ICI responses. D) Representative real‐time sensograms obtained from the Tr‐Apt13–DP‐SIS sensor following injection of BALF exosomes from a responder and a non‐responder. E) Non‐responders exhibiting significantly higher PD‐L1⁺ exosome levels, as detected at lower exosome concentrations. F) Corresponding dΨ from the Tr‐Apt13 sensor at 10^3^ EVs mL^−1^, confirming a stronger signal in non‐responders. G) ELISA‐based PD‐L1 quantification in BALF exosomes to discriminate non‐responders from responders. H) Real‐time sensograms obtained from DP‐SIS sensor functionalized with aPD‐L1. I,J) aPD‐L1–functionalized sensor also showing elevated PD‐L1⁺ exosomes in non‐responders, with statistical significance weaker compared to Tr‐Apt13. K) Correlation between dΨ obtained from aptamer‐ and antibody‐functionalized sensors. L–O) KM plots showing PFS of patients stratified by PD‐L1 IHC TPS, ELISA‐based exosomal PD‐L1, aPD‐L1–DP‐SIS sensor signal, and Tr‐Apt13–DP‐SIS sensor signal. Note that the significance levels are indicated as ^ns^ *p* ≥ 0.10, ^*^ *p* < 0.05, and ^**^ *p* < 0.01, determined by two‐sided Student's *t*‐tests.

The clinical utility of PD‐L1⁺ exosome detection using the Tr‐Apt13–functionalized DP‐SIS sensor was evaluated using BALF samples collected from eleven stage IV lung cancer patients prior to ICI therapy (Table S3, Supporting Information). CT imaging was performed before and after treatment (**Figure** **5**A), with seven patients classified as responders showing complete or partial responses, and four as non‐responders exhibiting stable or progressive diseases. PD‐L1 tumor proportion score (TPS) was simultaneously assessed using SP263 IHC assay from the tissue samples obtained prior to the therapy (Figure 5B). As shown in Figure 5C, conventional tissue PD‐L1 TPS failed to predict ICI response, with no significant difference observed between responders and non‐responders (40.1 ± 43.9% vs 32.8 ± 37.5%; *p* = 0.785).

BALF samples proximal to tumor sites were collected, and exosomes were isolated using the ExoQuick precipitation method following the manufacturer's protocol. The isolated exosomes were resuspended in 250 µL of PBS, and their concentrations were quantified by NTA (Figure S15, Supporting Information). Exosomes from each patient sample were subsequently diluted to the desired concentrations before introduction into the Tr‐Apt13–functionalized DP‐SIS sensor. Figure 5D presents representative sensogram signals obtained from a responder and a non‐responder following the injection of BALF‐derived exosomes at concentrations ranging from 10^1^ to 10^8^ particles mL^−1^. Non‐responders exhibited detectable levels of PD‐L1⁺ exosomes at a total exosome concentration of ≈5.50 × 10^2^ particles mL^−1^, which was significantly lower than the concentration required for detection in responders (≈4.60 × 10^4^ particles mL^−1^; *p* = 0.0053) (Figure 5E). Specifically, at a total exosome concentration of 10^3^ particles mL^−1^, dΨ for non‐responders and responders were 0.0053 ± 0.0024 and 0.0009 ± 0.0009, respectively (*p* = 0.0283), indicating high discriminatory capability of the sensor platform in estimating patient response to ICI therapy (Figure 5F).

For comparison, conventional ELISA was also employed to assess exosomal PD‐L1 expression at a concentration of 10⁶ particles mL^−1^. Despite exhibiting a moderate correlation with DP‐SIS sensor signals (Figure S16, Supporting Information), it failed to reliably predict ICI responses because PD‐L1 levels in most patient samples were close to the lower detection threshold (4.69 pg mL^−1^) (Figure 5G). These findings highlight that the ultrahigh sensitivity and enhanced resolution of the Tr‐Apt13–functionalized DP‐SIS sensor enable accurate prediction of therapeutic response based on PD‐L1⁺ exosome abundance, a capability that conventional assays cannot achieve.

Similarly, PD‐L1⁺ exosomes were detected using the same DP‐SIS sensor but functionalized with aPD‐L1 in place of Tr‐Apt13 (Figure 5H). Although the aPD‐L1–immobilized sensor also revealed elevated PD‐L1⁺ exosome signals in non‐responders, the difference between groups was less significant. Specifically, the signals detected from non‐responders and responders using aPD‐L1–functionalized sensor were 0.0052 ± 0.0029 and 0.0015 ± 0.0008, respectively (Figure 5I,J). While the signals obtained from aptamer‐ and antibody‐functionalized sensors were strongly correlated (r = 0.843; *p* = 0.001) (Figure 5K), the superior sensitivity of the Tr‐Apt13–functionalized DP‐SIS sensor—attributable to aptamer truncation, favorable orientation for PD‐L1⁺ exosome binding, and high immobilization density—enabled more accurate differentiation of ICI non‐responders based on PD‐L1⁺ exosome abundance. Furthermore, to compare our platform against existing label‐free biosensing methods,^[^ ^56^, ^57^ ^]^ we tested BALF‐derived exosomes (*n* = 5; 3 non‐responders and 2 responders) at concentrations between 10⁶ and 10⁸ particles mL^−1^ using an aPD‐L1–immobilized BLI sensor (Figure S17, Supporting Information). Only one non‐responder sample produced a detectable signal at the highest concentration (10⁸ particles mL^−1^), revealing the markedly higher sensitivity of the DP‐SIS system.

PFS was evaluated using multiple parameters assessed in this study, including tissue PD‐L1 TPS (Figure 5L), exosomal PD‐L1 expression measured by ELISA (Figure 5M), and PD‐L1⁺ exosome quantification obtained from DP‐SIS sensors functionalized with either aPD‐L1 or Tr‐Apt13 (Figure 5N,O). When patients were stratified based on the median value of each parameter, neither tissue PD‐L1 TPS nor exosomal PD‐L1 levels determined by ELISA showed prognostic significance for PFS. In contrast, quantification of PD‐L1⁺ exosomes using the DP‐SIS sensor revealed that patients with higher baseline levels had significantly shorter PFS, with the Tr‐Apt13–functionalized sensor yielding superior prognostic discrimination (14.05 ± 6.91 months vs 5.36 ± 6.50 months; *p* = 0.035). These results demonstrate the clinical potential of Tr‐Apt13–functionalized DP‐SIS sensing for predicting ICI efficacy and stratifying patient prognosis based on PD‐L1⁺ exosome levels in BALF.

## 3 Discussion

This study demonstrates a multi‐level engineering approach for ultrasensitive and clinically relevant detection of PD‐L1⁺ exosomes by integrating chemical aptamer optimization, mechanical sensing enhancements, and clinically relevant sampling strategies. At the chemical level, truncation of the SELEX‐derived Apt13 minimized non‐functional regions while preserving a thermodynamically stable stem‐loop structure, yielding Tr‐Apt13 with improved surface density and multivalent binding capability (Figures 2 and 3). Mechanically, incorporation of Tr‐Apt13 into the DP‐SIS sensor enabled real‐time, label‐free detection of PD‐L1⁺ exosomes with exceptional sensitivity, achieving a LOD of 9.8 × 10^0^ particles mL^−1^, while maintaining high specificity and precision (Figure 4). Clinically, the application of this platform to BALF samples from lung cancer patients enabled accurate discrimination between ICI responders and non‐responders, outperforming tissue PD‐L1 IHC and conventional ELISA in predictive sensitivity and prognostic stratification (Figure 5).

In the broader context of biomarker‐based prediction of ICI responses, multiple analytes such as tumor mutational burden (TMB), circulating tumor DNA (ctDNA), soluble PD‐L1, and exosomal PD‐L1 have been investigated using platforms including NGS, ddPCR, and ELISA (Table S4, Supporting Information).^[^ ^58^, ^59^, ^60^, ^61^, ^62^, ^63^, ^64^, ^65^ ^]^ Although these markers have shown the potential for clinical translation, the Tr‐Apt13/DP‐SIS system offers a distinct advantage by enabling direct and ultrasensitive detection of tumor‐proximal, exosome‐derived PD‐L1 without the need for a lysis step, thereby providing a straightforward readout of the tumor–immune interface. This platform may complement existing approaches within the broader biomarker landscape. Nevertheless, the present study was conducted with a limited number of clinical samples, and further validation in larger cohorts will be required to confirm its clinical applicability.

**Figure 6:**
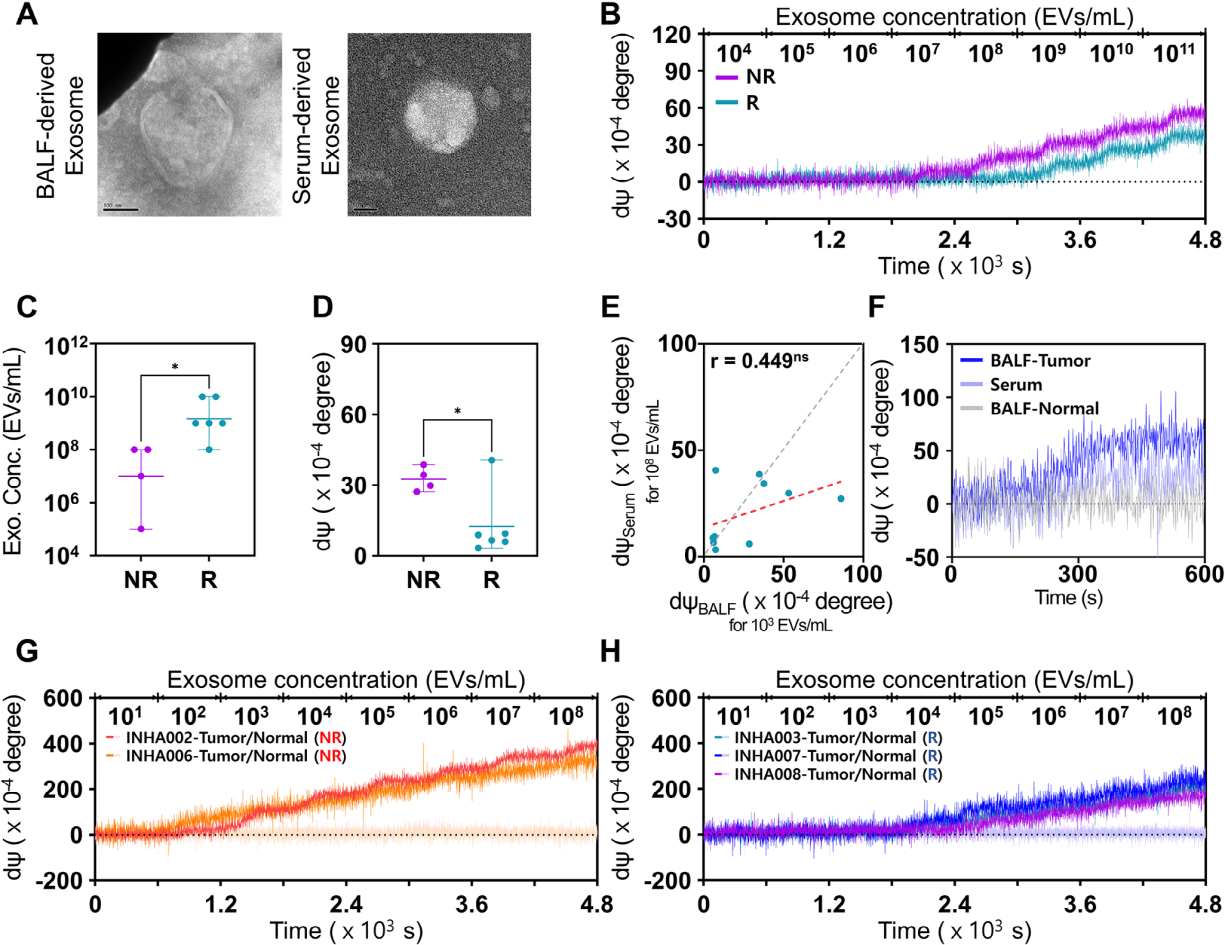
Comparative analysis of BALF‐ and serum‐derived exosomes for PD‐L1 detection using the DP‐SIS sensing platform. A) Representative TEM images of exosomes isolated from BALF and serum (scale bars: 100 nm for BALF‐derived exosomes and 50 nm for serum‐derived exosomes). B) Real‐time sensograms showing dΨ changes for BALF‐ and serum‐derived exosomes between responders and non‐responders C,D) Quantitative analysis of total exosome concentration required for the detection from serum‐derived exosomes and corresponding dΨ at the total exosome concentration of 10^8^ EVs mL^−1^ in responders and non‐responders. E) Correlation analysis of dΨ obtained from BALF‐ and serum‐derived exosomes. F) Validation of Tumor‐Derived PD‐L1⁺ Exosomes from Patient INHA004. BALF‐derived exosomes collected from tumor‐proximal regions exhibited stronger signals than those from serum, whereas BALF from non‐tumor regions showed no detectable signal. G,H) Real‐time detection of BALF‐derived exosomes from tumor‐proximal and non‐tumor regions using the Tr‐Apt13–functionalized DP‐SIS sensor. (G) shows data from two non‐responders, and (H) from three responders. Exosomes from non‐tumor regions exhibited no detectable PD‐L1⁺ signals across all five patients. Note that the significance levels are indicated as ^ns^ *p* ≥ 0.10 and ^*^ *p* < 0.05, determined by two‐sided Student's *t*‐tests.

BALF has been investigated as a clinically relevant medium for the diagnosis and monitoring of lung‐associated diseases due to its proximity to the disease site and its ability to capture biomolecular changes within the local tumor microenvironment.^[^ ^66^ ^]^ To assess whether BALF actually offers superior diagnostic resolution compared to serum, we conducted parallel experiments using serum‐derived exosomes from the same patient cohort (**Figure** **6**A; Figure S15, Supporting Information). Representative real‐time sensograms obtained from serum‐derived exosomes are shown in Figure 6B. Although serum‐derived exosomes also demonstrated significant differences between responders and non‐responders, the total exosome concentration required for detectable PD‐L1⁺ exosome signals was substantially higher (Figure 6C). Specifically, significant discrimination between responders and non‐responders was observed at 10⁸ particles mL^−1^ (0.0033 ± 0.0005 for non‐responders and 0.0012 ± 0.0014 for responders; *p* = 0.016), which is markedly greater than the threshold required for BALF‐derived exosomes (10^3^ particles mL^−1^) (Figures 6D and 5E). While dΨ values from BALF‐derived exosomes at 10^3^ particles mL^−1^ and plasma‐derived exosomes at 10⁸ particles mL^−1^ showed moderate correlation (r = 0.449; *p* = 0.193) (Figure 6E), the markedly lower detection threshold in BALF indicates its superior specificity and reduced background noise.

To further confirm that BALF contains a higher proportion of tumor‐derived PD‐L1⁺ exosomes, exosomes were injected at a concentration of 10⁸ particles mL^−1^ into the DP‐SIS sensor functionalized with exosome‐specific anti‐CD81 antibodies, followed by the injection of aPD‐L1 (1 µg mL^−1^). Notably, BALF‐derived exosomes from patient INHA004 produced a stronger signal than serum‐derived exosomes (Figure 6F). In contrast, exosomes isolated from BALF collected adjacent to non‐tumor tissue showed no detectable signal under the same conditions. Consistently, BALF‐derived exosomes from non‐tumor regions across all five patients tested also produced no detectable signal when analyzed with the Tr‐Apt13–functionalized DP‐SIS platform (Figure 6G,H). These findings demonstrate that tumor‐proximal BALF is substantially enriched in PD‐L1⁺ exosomes, supporting its potential as a highly specific source for assessing tumor‐derived exosomal biomarkers. However, it should be noted that using BALF as a biofluid has limitations. Its collection requires an invasive bronchoscopy procedure, which may restrict routine applicability compared to more accessible biofluids such as blood. Despite these limitations, the results presented in this study indicate that the superior specificity and reduced background noise of BALF‐derived exosomes enable their potential application for accurate prediction of ICI efficacy in lung cancer patients.

The feasibility of directly detecting PD‐L1⁺ exosomes in unprocessed BALF was also explored (Figure S18, Supporting Information). Due to the presence of abundant impurities that may induce non‐specific interactions on the sensor surface, and possibly the contribution of soluble PD‐L1, direct BALF analysis yielded stronger signals than those obtained from purified exosomes. Although this preliminary test was limited to three samples, BALF from non‐responders consistently produced higher signals than that from a responder. However, it should be noted that further clinical studies will be needed to clarify the proportion of signal attributable to exosomes versus non‐exosomal components.

The overall diagnostic performance of each method employed in this study is summarized in Figure S19 (Supporting Information). Although tested with a limited number of samples, the Tr‐Apt13–immobilized DP‐SIS sensor combined with BALF samples achieved both high sensitivity and specificity. At a total exosome concentration of 10^3^ particles mL^−1^, the highest signal among responders remained lower than the lowest signal among non‐responders, yielding an area under the receiver operating characteristic curve (AUC‐ROC) of 1.000 (*p* = 0.006). This performance highlights the synergistic advantages of aptamer truncation, which enabled dense immobilization and multivalent binding for ultrasensitive PD‐L1⁺ exosome detection, together with the use of tumor‐proximal BALF, which reduced background noise and enhanced specificity compared to serum. Importantly, the platform outperformed conventional ELISA and tissue PD‐L1 TPS in discriminating non‐responders, demonstrating its potential as a robust approach for predicting ICI efficacy.

## 4 Conclusion

This study establishes a multi‐level engineering strategy for ultrasensitive and clinically relevant detection of PD‐L1⁺ exosomes by combining aptamer truncation, sensor platform optimization, and clinically relevant sample selection. From computational modeling to molecular interaction measurements and in vitro affinity assays, we systematically validated the optimized truncated aptamer (Tr‐Apt13), which demonstrated enhanced targeting of PD‐L1‐expressing biomolecules compared to conventional antibodies, attributable to its superior multivalent binding capability enabled by high surface immobilization density and controlled orientation. Integration of Tr‐Apt13 into the DP‐SIS sensor enabled real‐time, label‐free detection of PD‐L1⁺ exosomes with a detection limit as low as 9.8 × 10⁰ particles mL^−1^, outperforming antibody‐based surfaces and ELISA assays in both sensitivity and specificity. Clinically, analysis of PD‐L1⁺ exosomes from BALF samples demonstrated accurate discrimination between ICI responders and non‐responders and superior prognostic stratification compared with tissue PD‐L1 IHC, as well as conventional antibody assays. BALF‐derived exosomes also exhibited markedly higher tumor specificity, as exosomes from tumor‐proximal BALF showed significantly stronger PD‐L1⁺ exosome signals compared to those obtained from serum. Taken together, these findings support the use of the Tr‐Apt13–functionalized DP‐SIS platform for PD‐L1⁺ exosome detection as a promising liquid biopsy approach for precise prediction of immunotherapy efficacy and improved patient stratification in lung cancer.

## 5 Experimental Section

### Statistics

Statistical analyses were performed using IBM SPSS Statistics (version 29; IBM Corp., Armonk, NY). Continuous variables, such as surface wettability, in vitro targeting efficacy measured using cell retention assay, and PD‐L1 expression obtained from ELISA were compared using two‐sided Student's *t*‐test (*n* ≥ 3 for all experiments). Continuous variables, including surface wettability, in vitro targeting efficacy from cell retention assays, and PD‐L1 expression measured by ELISA, were compared using two‐sided Student's *t*‐tests (*n* ≥ 3 for all experiments). For clinical sample analyses, tissue PD‐L1 TPS, exosomal PD‐L1 levels measured by ELISA, and PD‐L1⁺ exosomes detected using antibody‐ or aptamer‐immobilized DP‐SIS sensors were compared between responders and non‐responders using two‐sided Student's *t*‐tests. Correlations between these findings were assessed using Pearson's correlation analysis, and the significance levels are given in a two‐sided analysis. Results presented in this study were given as mean ± standard deviation unless otherwise noted. Statistical significance was defined as ^ns^ *p* ≥ 0.10, ^#^ *p* < 0.10, ^*^ *p* < 0.05, ^**^ *p* < 0.01, and ^***^ *p* < 0.001.

### Ethical Approval and Informed Consent

All study procedures and sample collections were approved by the Institutional Review Board of Inha University Hospital (IRB No. 2005‐03‐001), and written informed consent was obtained from all participants.

Other detailed experimental procedures are provided in the Supporting Information.

## Conflict of Interest

Hyun Mo Cho is the founder and CEO of SIS Sensor Corporation, which is in the process of commercializing the technology described in this manuscript. This company may have a potential financial interest in the publication of this research. The other authors declare no competing interests.

## Supporting information



Supporting Information

## Data Availability

The data that support the findings of this study are available from the corresponding author upon reasonable request.
