# Supplementary material for: Aptamer‐Engineered Ellipsometry for Clinical Detection of BALF‐Derived Exosomes: Multi‐Level Engineering for Prognostic Evaluation of Immunotherapy Responses
Source: Adv Sci (Weinh). 2025 Oct 31;13(3):e15247. doi: 10.1002/advs.202515247 (PMC12806343; doi:10.1002/advs.202515247)
Supplement: Supplementary file 1 — Supporting Information [file ADVS-13-e15247-s001.pdf]

Supporting Information

Aptamer-Engineered Ellipsometry for Clinical Detection of BALF-Derived Exosomes: Multi-level Engineering for Prognostic Evaluation of Immunotherapy Responses

Euna Jeong†, Jung Hyun Choi†, Minyoung Lee†, Jun Hyoek Lim†, Seokho Jung, Yeeun Woo, Joo-young Kim, Jaehyeon Ok, Lucia Kim, Woo-jin Jeong, Won Chegal, Hyun Mo Cho, Chulhwan Park, Jiyoon Bu, Dong Hyung Kim*, Taek Lee**

Experimental Section

Materials: Human PD-L1 protein was purchased from Sino Biological Inc. (Beijing, China). The nonspecific proteins, such as albumin and myoglobin which were generally a component of human serum, and amicon ultra centrifugal filter 3K were purchased from Sigma-Aldrich (St. Louis, MO). EZ-Link Sulfo-NHS-LC-Biotin were purchased from Thermo Fisher Scientific (Waltham, MA). Streptavidin magnet beads were purchased from Genscript (Piscataway, NJ, USA). XELEX DNA Kit was purchased from EURx (Gdańsk, Poland). PCR master mix and 100 bp DNA Ladder were purchased from Bioneer (Daejeon, Korea). Dyne LoadingSTAR was purchased from DyneBio (Seongnam, South Korea). DNA sequence identification with high binding specificity was analyzed by Solgent (Daejeon, Korea). PD-L1 aptamer modified with Amine functional group (-NH_2) at the 5′ end was synthesized by Bionics (Seoul, Korea). Additional details on specific materials are provided in the corresponding subsections.

SELEX Process and Binding Analysis for Identification of PD-L1-Specific Aptamer: PD-L1 protein was initially biotinylated to facilitate binding to streptavidin-coated magnetic beads. 2 mM Sulfo-NHS-LC-Biotin was added to 0.25 mg/mL of human PD-L1 protein and incubated for 1 hour. Non-biotinylated material was removed by centrifugation. The solution was diluted in 1 × phosphate-buffered saline (PBS) and transferred to an amicon ultra centrifugal filter 3K. It was then centrifuged at 12,000 rpm. The biotinylated PD-L1 protein was incubated with streptavidin magnetic beads for 1 hour. Unbound material was removed by washing with 1 × SELEX buffer more than 3 times. A random DNA library and 1 × SELEX buffer were mixed with the PD-L1 bead complex and incubated for 1 hour to facilitate the binding of high-affinity DNA sequences to PD-L1. Unbound DNA was removed through the same washing process mentioned above. DNA selectively bound to PD-L1 was eluted from the beads by heat denaturation at 85 ℃. The obtained DNA sequence was amplified using polymerase chain reaction (PCR) using iCycler (Bio-Rad, California). The PCR reaction was performed with the obtained DNA, Bank40-5′, Bank40-3′, and PCR master mix. The reaction conditions were 95 ℃ for 2 minutes and 30 seconds, 55 ℃ for 1 minute, 72 ℃ for 3 minutes, and 70 ℃ for 5 minutes, repeated 13 times. The amplified nucleic acids were continuously utilized in the next round of SELEX. The entire SELEX cycle was performed identically 10 times to gradually select high-affinity sequences. Following completion of 10 SELEX cycles, the last product was sequenced to identify DNA sequences with high binding specificity. Among the 50 candidate

sequences identified, those exhibiting the highest binding affinity were further analyzed and selected.

Truncation Strategy for Optimizing Aptamer Function: A truncation strategy was applied to PD-L1 Apt13, which was verified to have the best binding specificity among the selected sequences. This strategy gradually removes primer regions that are not involved in binding to the target. This not only enhances cost-effectiveness but also preserves the core stem-loop structure of the aptamer, and can further improve binding affinity.

Computer-Aided Binding Precition Modeling: Aptamer docking simulation analysis was additionally performed on the truncated PD-L1 Tr-Apt13 to visualize and evaluate the binding affinity. The 3D structure information of the PD-L1 protein was obtained from the AlphaFold Protein Structure Database (https://alphafold.ebi.ac.uk/) and the RCSB PDB database (https://www.rcsb.org/). The obtained protein 3D structure and aptamer sequence were analyzed using the HDOCK web server (https://hdock.phys.hust.edu.cn/), and all docking simulations were performed based on the default parameter values. The docking model with the best score was precisely analyzed for noncovalent interactions using the Protein-Ligand Interaction Profiler (PLIP, https://www.plip-tool.biotec.tu-dresden.de/plip-web/plip/index), and the final visualization was performed using PYMOL software.

Cell Preparation and Exosome Enrichment from Cell Lines: Human breast cancer cell line MDA-MB-231 (RRID: CVCL_0062) and human promyelocytic leukemia cells HL-60 (RRID: CVCL_0002) were used to obtain PD-L1^{High} and PD-L1^{Negative} exosomes, respectively. MDA-MB-231 cells were cultured as adherent monolayers in Dulbecco’s Modified Eagle Medium (DMEM; Gibco, Grand Island, NY) supplemented with 10% (v/v) fetal bovine serum (FBS; Gibco) and 1% (v/v) penicillin–streptomycin (P/S; Gibco). HL-60 cells were maintained in suspension in RPMI-1640 medium (Gibco) supplemented with 10% FBS and 1% P/S. All cultures were incubated at 37 ℃ in a humidified atmosphere containing 5% CO_2.

For cell retention assays, cells were stained with 2 μM calcein-AM (Thermo Fisher Scientific, San Jose, CA) in 4 mL of culture medium for 30 minutes at 37 °C, followed by washing via centrifugation at 1,300 rpm for 3 minutes and resuspension in 1 mL of fresh medium before introduction into the flow chamber.

For collecting exosomes from these cell lines, cells were initially seeded into T-175 flasks and allowed to reach 60–80% confluency. The medium was then replaced with exosome-free media. After 48 hours of incubation, the conditioned medium was collected and centrifuged twice at

2,000 rpm for 10 minutes to remove cells and large debris. The supernatant was concentrated using Amicon® Ultra Centrifugal Filters (10 kDa cutoff; Millipore, MA, USA) at 4,000 × g for 30 minutes and subsequently filtered through a 0.22 μm vacuum filter. Exosomes were then isolated using ExoQuick™ (System Biosciences, Palo Alto, CA) according to the manufacturer's instructions. Briefly, 1 mL of pre-filtered medium was mixed with 250 µL of ExoQuick™, vortexed, and incubated at 4 °C for 30 minutes. The mixture was centrifuged at 3,000 × g for 30 minutes, and the resulting exosome pellet was gently resuspended in 1 × PBS. All steps were performed on ice or at 4 °C to preserve exosomal integrity. The final exosome suspension was adjusted to a total volume of 250 µL. Particle concentration and size distribution were then quantified using nanoparticle tracking analysis (ZetaView® PMX230; Particle Metrix, Meerbusch, Germany).

Biolayer interferometry (BLI) Analysis: Bio-layer interferometry experiments were performed on a Gator® Pilot system (GatorBio, USA) employing amine-reactive (AR) probes (SKU 160008). Activation, immobilization, blocking, association, and dissociation steps were all conducted in 1 × PBS (pH 7.4). AR probes were initially activated by immersion in 200 mM EDC/100 mM NHS for 300 s, followed by covalent coupling of human PD-L1 protein (295.9 nM). Unreacted sites were subsequently blocked with 1 M ethanolamine for 300 s, the probes were rinsed in the same buffer, and a baseline measurement was recorded. For direct binding assays, analytes—namely aptamer variants (Apt45, Apt13, and Tr-Apt13 at 12, 6, and 3 μM) or anti–PD-L1 antibody (40, 20, and 10 nM)—were introduced for 300 s association and 600 s dissociation. In reverse binding assays, AR probes were activated and immobilized with 5 μM of anti–PD-L1 antibody, Apt13, or Tr-Apt13, blocked as described above, and then exposed to recombinant PD-L1 (300 and 150 nM) for 300 s association followed by 600 s dissociation.

Surface Preparation for Cell Retention Assay: Epoxy-functionalized microscope slides (Tekdon Incorporated, Myakka City, FL) were immobilized with aptamers or antibodies using an 8-well silicon gasket. Briefly, carboxyl groups were introduced onto epoxy glass slides by incubating the slides with glycine (1 mg/mL) for 12 hours. Subsequently, aptamers (5 nM) or anti–PD-L1 antibodies (Cat# SIM0009, Bio × Cell, San Antonio, TX) were immobilized onto the surface via amine coupling, consisting of a 20-minute incubation with EDC (3.6 mg/mL) and NHS (5.6 mg/mL) (Thermo Fisher Scientific, San Jose, CA). The surface was further blocked with methyl amine to reduce the non-specific bindings using same EDC/NHS chemistry.

Cell Retention Assay for In Vitro Affinity Assessment: Antibody- or aptamer-functionalized slides were assembled into custom-designed flow chambers (**Figure S3**). Fluorescently labeled cells were introduced into the chamber using a syringe pump at an initial flow rate of 500 μL/min and allowed to adhere under static conditions for 10 minutes at room temperature. Cell retention was then evaluated by sequential washing at increasing flow rates, followed by fluorescence imaging with an inverted microscope (Zeiss Axiocam 705, Carl Zeiss, Germany). The washing protocol included two 10-minute washes at 50 μL/min and 100 μL/min, respectively, and a final 1-minute wash at 1,000 μL/min.

Deglycosylation of MDA-MB-231 Cells: To reduce N-glycans at the cell surface, specifically from membrane-bound PD-L1, MDA-MB-231 cells were treated with PNGase F (50 U/mL; Enzynomics, Daejeon, Korea). Briefly, 1 × 10^6 cells were incubated with 1 mL of culture medium and 1 mL of PNGase F solution at 37 °C in a humidified atmosphere containing 5% CO_2 for 3 h. Following the treatment, PNGase F–treated cells were subjected to a retention assay on Apt45-immobilized surfaces under the same conditions as untreated controls. Additionally, cell viability after PNGase F treatment was assessed using a two-color live/dead assay (Thermo Fisher Scientific) according to the manufacturer's protocol. Briefly, cells were incubated with a staining solution containing 4 μM calcein and 4 μM ethidium homodimer for 10 min, followed by washing with fresh medium. Viability was evaluated relative to untreated control cells.

SIS Sensor Chip Fabrication: n-type silicon wafers (11.55 mm × 11.55 mm) were pretreated by sequential rinsing with acetone and ethanol to remove organic residues, with nitrogen gas used to blow-dry the surfaces between steps. Wafers were then subjected to piranha solution treatment (3:1 v/v sulfuric acid to hydrogen peroxide) to eliminate remaining contaminants and enhance surface hydroxylation. For silanization, wafers were incubated in 2% (v/v) APTES (3-aminopropyltriethoxysilane) in anhydrous ethanol for 12 hours at room temperature. After incubation, wafers were thoroughly rinsed with anhydrous ethanol, baked at 70 °C for 30 minutes, and mounted onto a dual-prism assembly to fabricate the DP-SIS (dual prism–solution-immersed silicon) sensor.

For receptor immobilization, either anti–PD-L1 antibodies (Cat# SIM0009, Bio × Cell, San Antonio, TX) or aptamers were covalently conjugated to the silanized surface at a concentration of 10 nmol/mL via NHS ester chemistry. Unreacted active sites were subsequently blocked with

0.5% (w/v) BSA (Sigma-Aldrich, St. Louis, MO) in PBS. All immobilization steps were performed under continuous flow at 30 μL/min.

Contact Angle Measurement: Contact angle measurements were conducted to assess changes in surface wettability after immobilizing aptamers or anbitodies, using a Phoenix 300 Touch contact angle goniometer (SEO, Suwon, Korea). A 20 μL droplet of deionized water (ddH_2O) was applied to bare n-type silicon wafers, anti–PD-L1–immobilized surfaces, and Tr-Apt13–immobilized surfaces via the sessile drop method. Static contact angles were recorded, and droplet images were analyzed using ImageJ software (NIH, Bethesda, MD).

X-ray photoelectron spectroscopy Analysis: X-ray photoelectron spectroscopy (XPS) was used to analyze the surface chemical composition and functional groups on bare, anti–PD-L1–functionalized, and Tr-Apt13–functionalized n-type silicon wafers. Measurements were conducted using a NEXSA XPS system (Thermo Scientific, Waltham, MA) equipped with a monochromatic Al Kα X-ray source (1486.6 eV). Spectra were acquired using a 400 μm spot size, with a pass energy of 200 eV for wide scans to determine elemental composition and 50 eV for high-resolution scans to identify specific chemical states. Core-level spectra for C 1s, N 1s, O 1s, and Si 2p were collected to evaluate surface modifications, including changes in silicon oxide levels and functional group incorporation associated with antibody and aptamer immobilization.

Surface Roughness Measurement Using AFM: Surface roughness of bare silicon wafers, anti–PD-L1–functionalized surfaces, and Tr-Apt13–functionalized surfaces was analyzed using atomic force microscopy (AFM; XE7, Park Systems, Suwon, Korea). Topographic images were obtained in tapping mode under ambient conditions with a multimode scanner and a Nanoscope V controller. Data acquisition and image processing were performed using XEI software (v6.36). Surface height profiles and root mean square roughness (Rq) values were calculated from horizontal line scans.

Nanoparticle Tracking Analysis: Nanoparticle tracking analysis (NTA) was performed using a ZetaView® PMX230 system (Particle Metrix, Meerbusch, Germany) in scatter mode to determine the concentration and size distribution of exosomes derived from both cell lines and patient samples, following the manufacturer’s protocol. Each sample was analyzed under standardized acquisition settings, and the mean particle diameter and concentration were

calculated using ZetaView software. All measurements were conducted in triplicate to ensure reproducibility and minimize technical variability.

Patient Recruitment and Clinical Sample Collection: A total of eleven patients with lung cancer were enrolled in this study. BALF samples were collected from tumor-proximal sites in all eleven patients and from adjacent normal tissue in five patients, all prior to the initiation of ICI therapy at Inha University Hospital. Specifically, during bronchoscopic examination, bronchial washing (BW) was performed on each patient by placing 30 mL sterile saline by wedging the bronchoscope at the subsegmental bronchus where the lung nodule or mass was thought to be located. In five of the eleven patients, an additional BW sample was obtained from the contralateral lung as a control using the same procedure. BW fluid was collected in centrifuge tubes, and centrifuged at 1,000 × g for 20 minutes at 4℃. The BW supernatants and precipitates were separated and stored at −80℃.

Concurrently, serum samples were collected from ten of the same lung cancer patients, also prior to ICI treatment. Five milliliters of whole blood were drawn into EDTA tubes and centrifuged at 1,000 × g for 20 minutes at 4 ℃, after which the resulting plasma fraction was aliquoted and stored at −80 °C.

Note that all study procedures and sample collections were approved by the Institutional Review Board of Inha University Hospital (IRB No. 2005-03-001), and written informed consent was obtained from all participants.

Tumor responses to ICI treatments were assessed according to the Response Evaluation Criteria in Solid Tumors (RECIST), version 1.1. Patients achieving partial response were classified as responders, while those with stable disease (SD) or progressive disease (PD) were classified as non-responders. PD-L1 TPS was determined by immunohistochemistry (IHC) on formalin-fixed, paraffin-embedded (FFPE) tumor sections using a validated anti–PD-L1 antibody (clone SP263; Roche Tissue Diagnostics, Oro Valley, AZ). TPS scoring was performed by a board-certified pathologist (L. Kim) based on SP263 staining patterns.

Exosome Enrichment from Clinical Samples: Exosomes were isolated from BALF or serum using the ExoQuick™ Plasma Prep and Exosome Precipitation Kit (System Biosciences, Palo Alto, CA) according to the manufacturer's instruction. Briefly, 250 μL of cell-depleted BALF or serum was mixed with 63 μL of ExoQuick™ solution, vortexed thoroughly, and incubated at 4 ℃ for 30 minutes to promote exosome precipitation. The mixture was then centrifuged at 1,500 × g for 30 minutes, and the resulting pellet was gently resuspended in 1 × PBS. All steps

were performed on ice or at 4 °C to preserve sample integrity. The resuspended exosomes were diluted to a final volume of 250 μL. Particle concentration was measured using nanoparticle tracking analysis as aforementioned.

Transmission Electron Microscopy Imaging: Transmission electron microscopy (TEM; CM200, PHILIPS, Amsterdam, Netherlands) was used to examine the morphology of exosomes isolated using ExoQuick™. For sample preparation, 2 μL of exosome suspension was placed onto a carbon-coated copper grid (200 mesh, Cu; Electron Microscopy Sciences, Hatfield, PA, USA) and air-dried at room temperature. Negative staining was performed with 2 μL of Uranyless EM stain (Electron Microscopy Sciences), a non-radioactive alternative to uranyl acetate. Excess stain was gently removed, and the grid was dried completely before imaging. TEM imaging was conducted using the CM200 system operated at 120 kV.

PD-L1 ELISA Assessment: A commercial human PD-L1 ELISA kit (Invitrogen, Waltham, MA) was used to quantify PD-L1 expression from cells or exosomes, following the manufacturer's instruction. MDA-MB-231 and HL-60 cells, along with their corresponding exosome samples, were lysed by mixing with an equal volume of 2 × radioimmunoprecipitation assay (RIPA) buffer (150 mM NaCl, 1% NP-40, 1% sodium deoxycholate, 0.1% SDS, 50 mM Tris-HCl, 2 mM EDTA, pH 7.5) supplemented with protease inhibitors (Protease Inhibitor Cocktail II, Sigma-Aldrich, St. Louis, MO). All procedures were performed on ice to preserve protein integrity. Lysates were applied to ELISA plates at a total protein concentration of 1 mg/mL.
For the detection of PD-L1 on exosomes obtained from clinical samples, BALF-derived exosomes were analyzed directly—without membrane lysis—using the same PD-L1 ELISA kit. Exosome derived from clinical samples were loaded onto ELISA plates at a concentration of 10^6 particles/mL. All assays were performed according to the manufacturer's protocol.

PD-L1$^+$ Exosome Quantification Using SIS Measurement: Exosome samples were prepared in PBS containing 0.05% (v/v) SDS (SDS-PBS) at concentrations of 10^1 to 10^8 particles/mL for cell line–derived (MDA-MB-231 and HL-60) and BALF-derived exosomes, and 10^5 to 10^{12} particles/mL for serum-derived exosomes. Concentrations were serially diluted on an exponential scale to allow direct concentration-dependent detection of PD-L1–expressing exosomes.
Samples were sequentially introduced onto sensor surfaces functionalized with anti–PD-L1 antibodies, Apt13, Apt45, or Tr-Apt13 at a constant flow rate of 100 μL/min. Binding

interactions were monitored in real time, with changes in the amplitude ratio (Ψ) reflecting receptor–exosome interactions. Binding signals appeared at approximately 200 s, reaching equilibrium around 400 s. Note that each measurement was recorded over 600 s for each concentration.

To verify surface PD-L1 expression on exosomes, 10^8 particles/mL of exosomes derived from serum, tumor-proximal BALF, and normal BALF were injected into SIS sensor chips functionalized with anti-CD81 antibodies (5 μg/mL). Following exosome capture, anti–PD-L1 antibodies (1 μg/mL; Cat# SIM0009, Bio × Cell, San Antonio, TX) were introduced into the sensor. All procedures were performed at a flow rate of 100 μL/min for 600 seconds.

Note S1. *Intuitieve interpretation of 2D structure of structure–function relationships in aptamers*: The 2D structure of an aptamer (**Figure 2A** and **Figure 2B**) allows for intuitive interpretation of structure–function relationships by visualizing the base pairing status and structural features of each nucleotide using color coding. The colors used in the diagram are categorized based on functional roles: red indicates unpaired bases, primarily located in loop regions, which are highly likely to be directly involved in interactions with the target molecule. Black represents stem regions where base pairs are formed, contributing to the stabilization of the double helix structure. Purple is used to highlight particularly thermodynamically stable base pairs within the stem, such as G–C pairs, which form three hydrogen bonds and confer greater stability than A–T pairs. While these stable stem structures lower the overall ΔG of the aptamer and thus enhance its thermodynamic stability, they do not necessarily correlate with binding specificity.

Green emphasizes functionally important unpaired bases, often located at junctions between stems or within loops, which provide structural flexibility and enable steric interactions with the target. Although such unpaired bases may be thermodynamically less favorable due to increased free energy, they can contribute to functional stability upon target binding through favorable molecular interactions. The numbers indicated in the image (e.g., 10, 20, 30, 40) refer to the position (index) of each base within the sequence and serve as reference points for structural analysis and tracking of binding sites. The 5′ and 3′ labels denote the directionality of the aptamer sequence, which is critical for interpreting its folding pathway and the order of base pair formation; the aptamer is generally predicted to fold from the 5′ end toward the 3′ end.

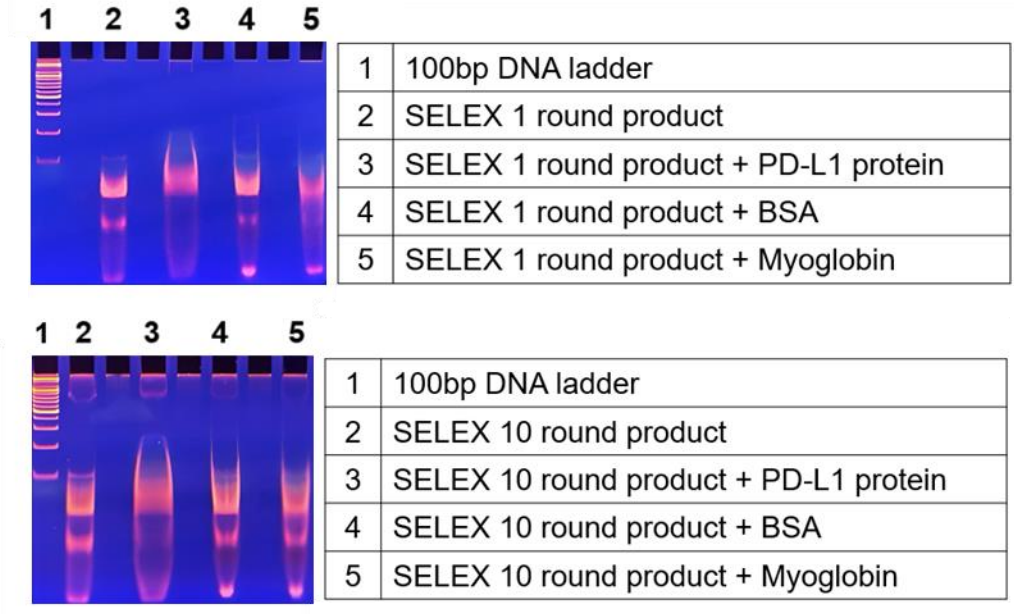

Figure S1. TBE-PAGE analysis of SELEX products after round 1 (top) and round 10 (bottom). The round 10 product demonstrates selective interaction with recombinant PD-L1, with negligible binding to control proteins, indicating successful enrichment through the iterative SELEX process.

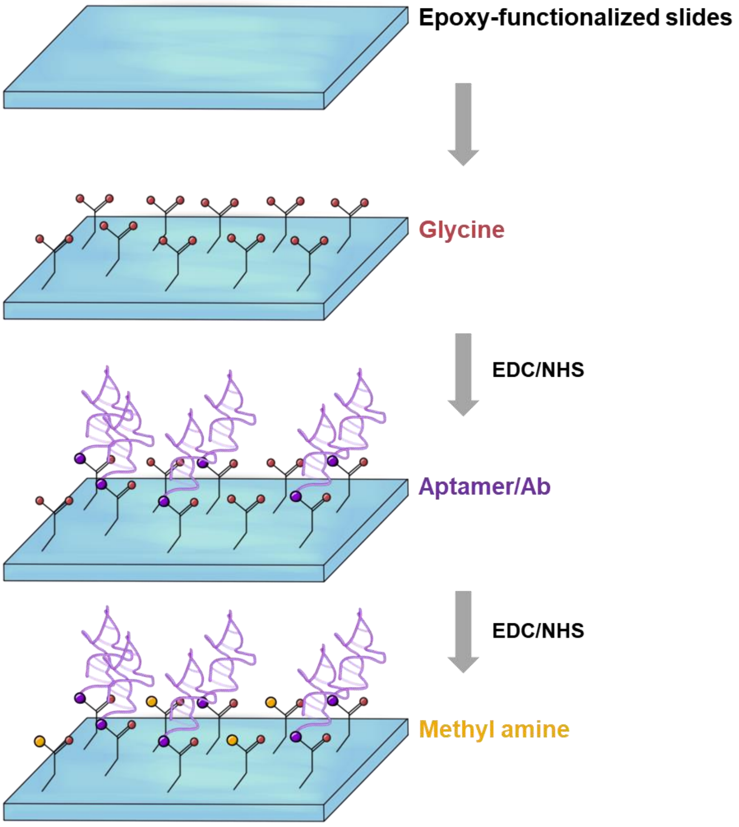

Figure S2. Schematic illustration of the surface functionalization process for the cell retention assay. Epoxy-functionalized microscope slides were initially incubated with glycine to introduce carboxyl groups. Aptamers or aPD-L1 were then immobilized via amine coupling, followed by methylization with methyl amine to reduce non-specific binding.

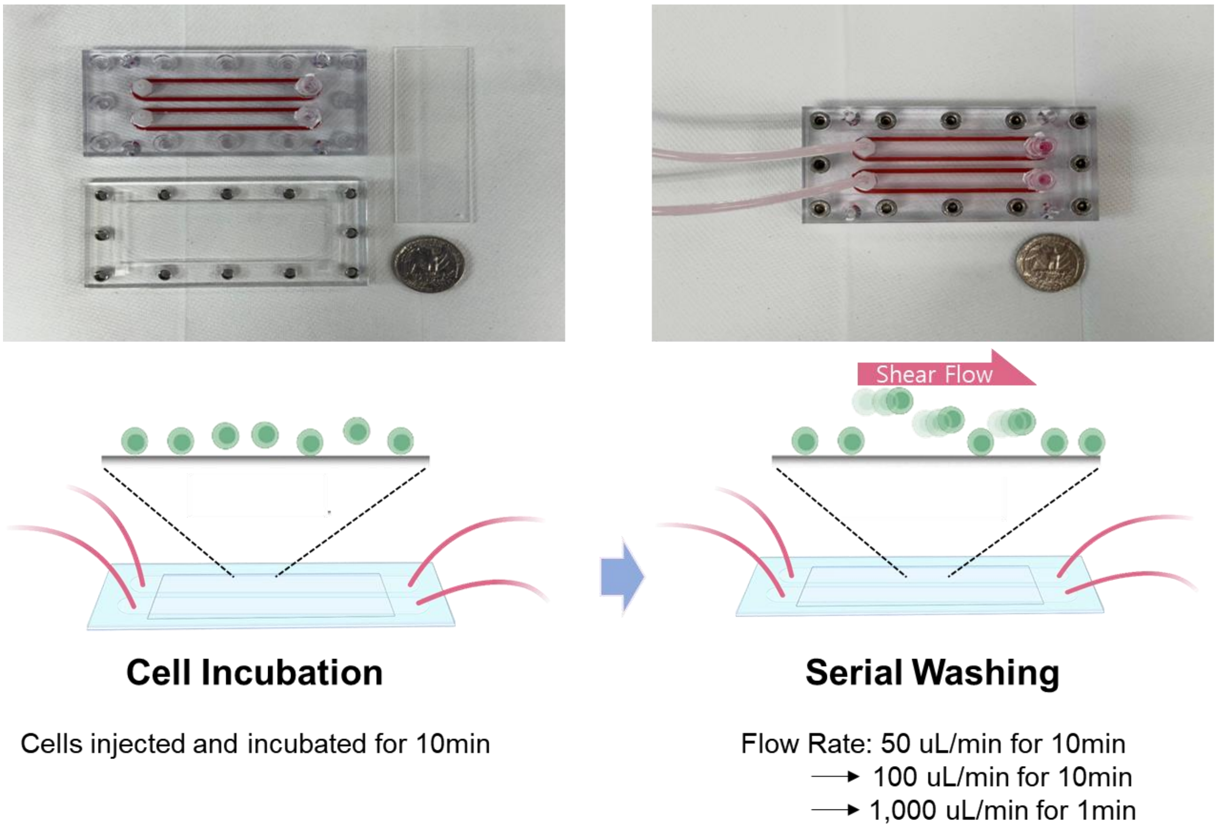

Figure S3. Custom-designed flow chamber setup and schematic of the cell retention assay. (Top) Images of the assembled flow chamber with aptamer- or antibody-functionalized slides. (Bottom) Schematic representation of the assay workflow: fluorescently labeled cells were introduced into the chamber under static conditions to allow adhesion, followed by sequential washing under increasing shear flow to assess cell retention on functionalized surfaces.

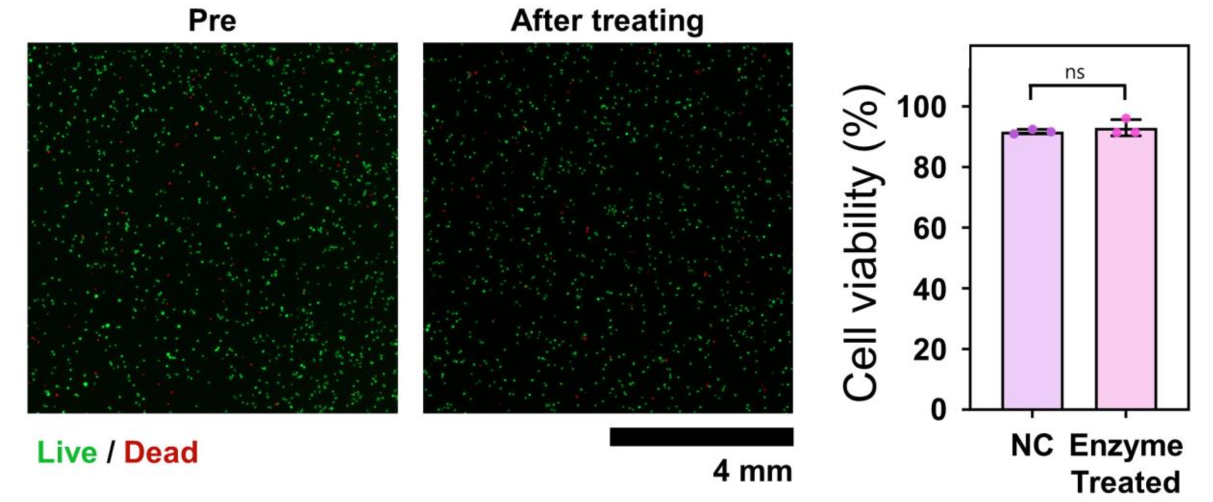

Figure S4. Cell viability measured using a two-color live/dead assay after PNGase F (50 U/mL) treatment for 3 h.

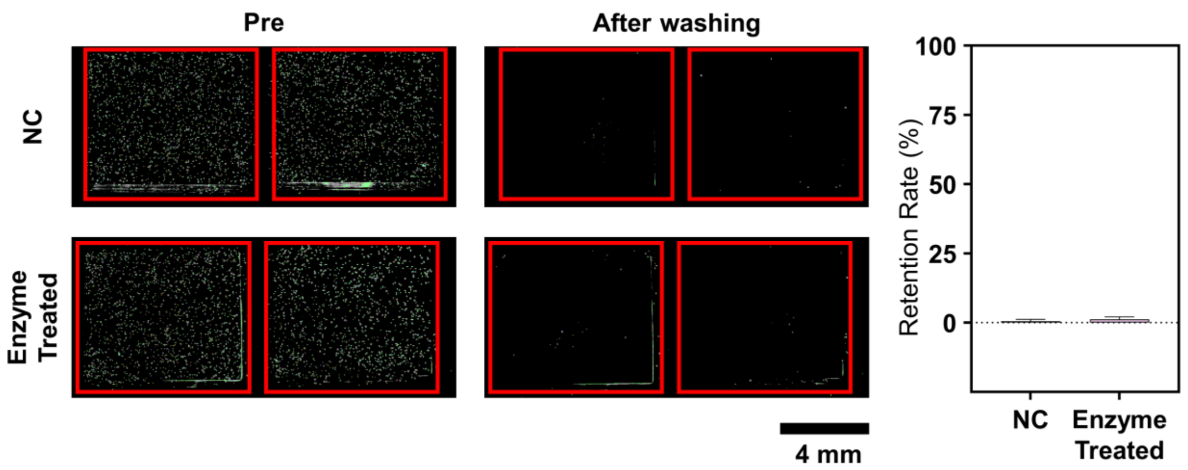

Figure S5. Effect of PNGase F treatment on the adhesion of PD-L1^{Negative} HL-60 cells to the Apt45-functionalized surface (n = 3, in duplicates). Cells were incubated in an eight-well chamber for 1 h, followed by washing.

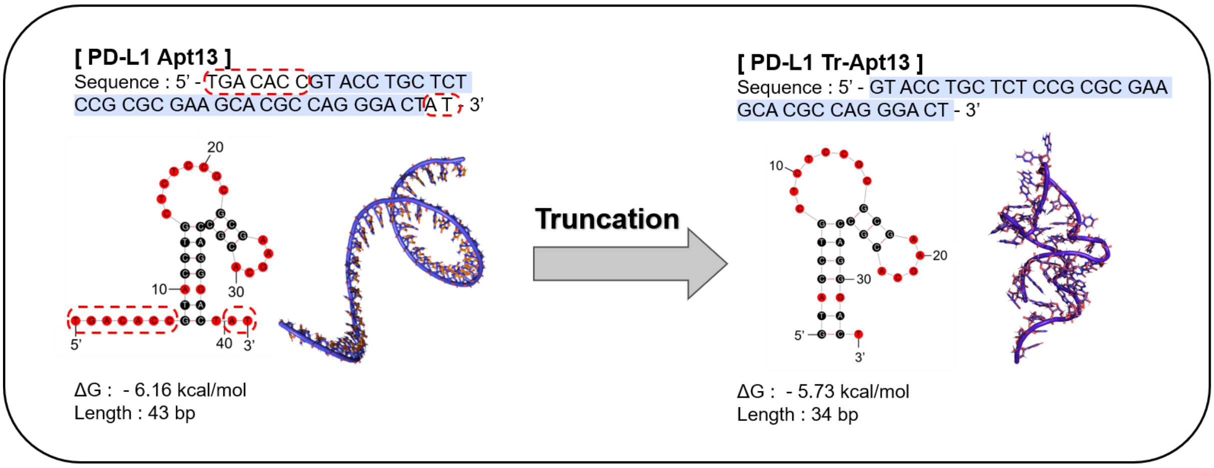

Figure S6. Truncation process of the PD-L1 aptamer for optimization of binding performance.

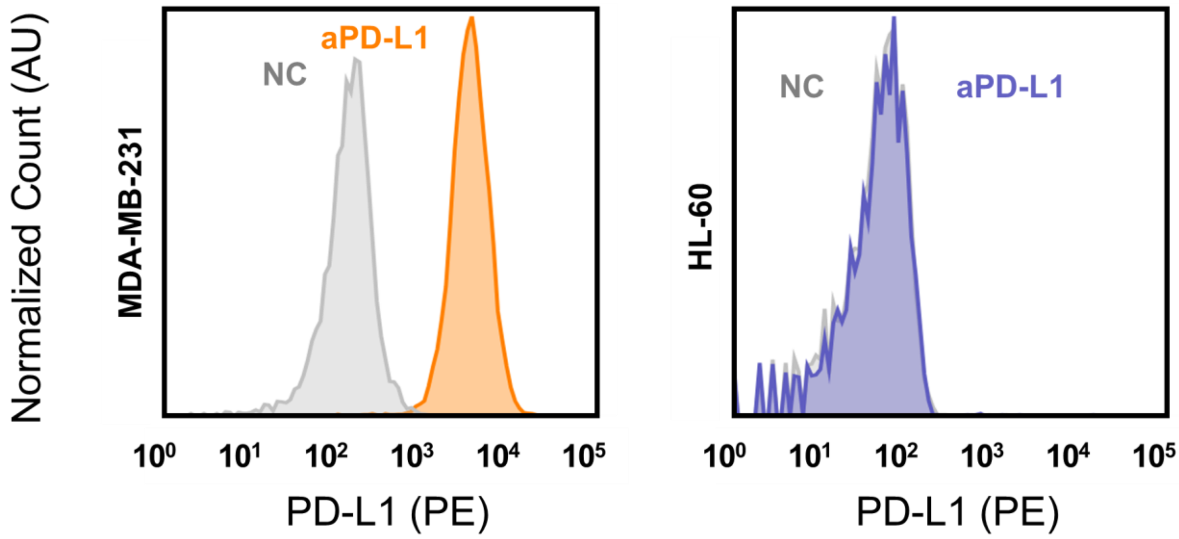

Figure S7. Flow cytometry analysis of PD-L1 expression in PD-L1^{High} MDA-MB-231 cells and PD-L1^{Negative} HL-60 cells.

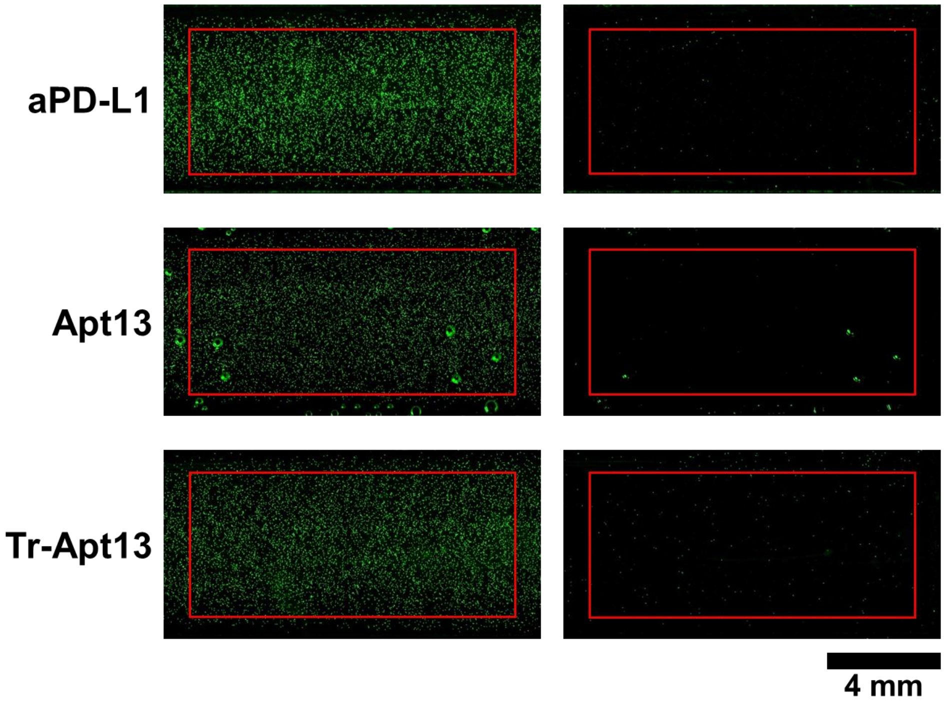

Figure S8. Representative fluorescence microscopy images of HL-60 cells following the cell retention assay on aptamer- or antibody-functionalized surfaces. Images were captured before (left) and after (right) sequential washing under increasing shear flow. Minimal cell retention was observed from all surfaces tested.

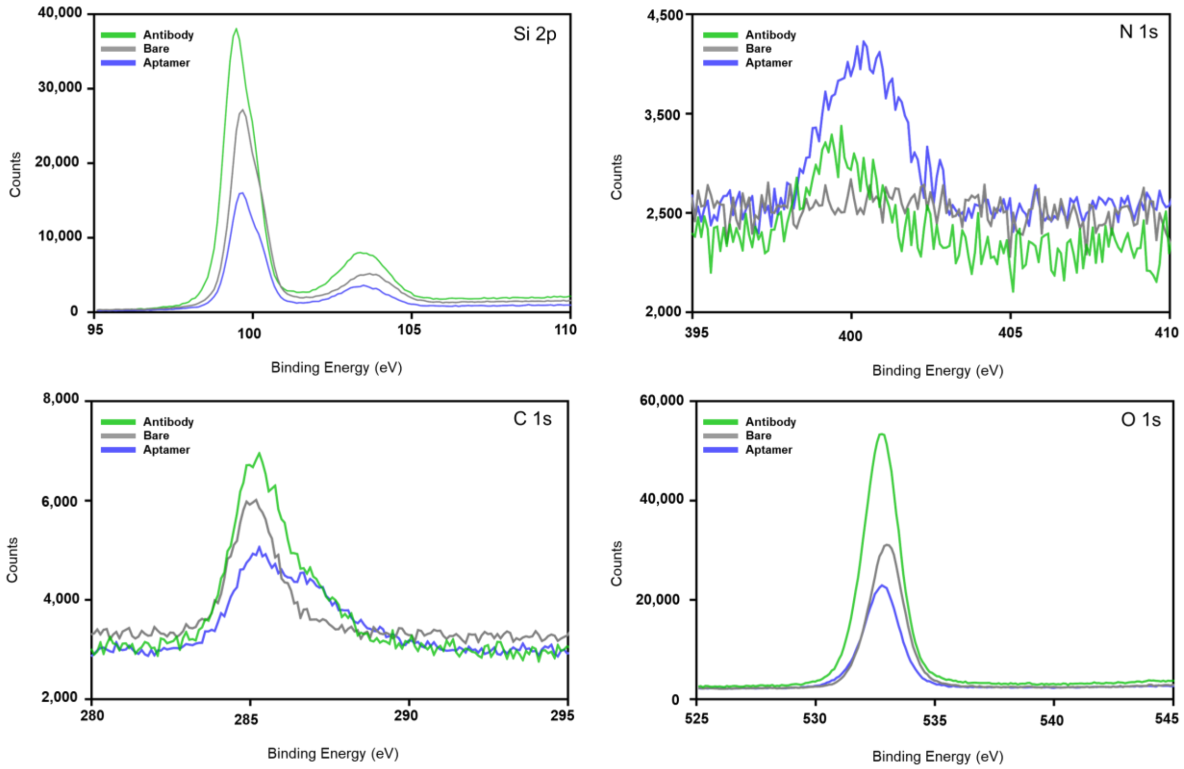

Figure S9. XPS spectra of Si 2p, N 1s, C 1s, and O 1s regions for bare silicon wafers, antibody–functionalized surfaces, and aptamer–functionalized surfaces. Notable changes in elemental composition, including increases in N 1s and C 1s signals, confirm successful immobilization of antibodies and aptamers on the silicon wafer surfaces.

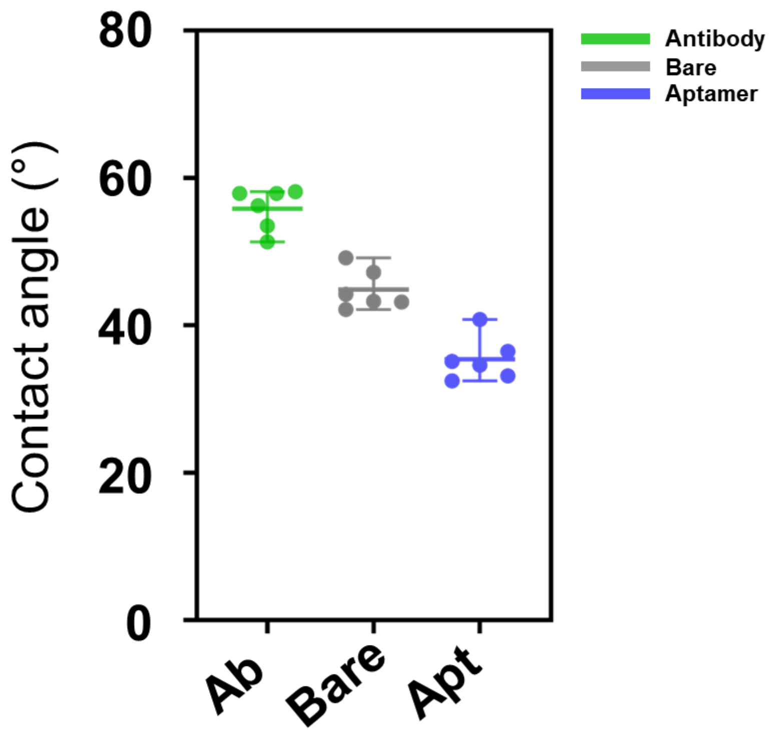

Figure S10. Contact angle measurements of bare silicon wafers, antibody–functionalized surfaces, and aptamer–functionalized surfaces. Aptamer functionalization increased surface hydrophilicity, whereas antibody functionalization resulted in greater surface hydrophobicity.

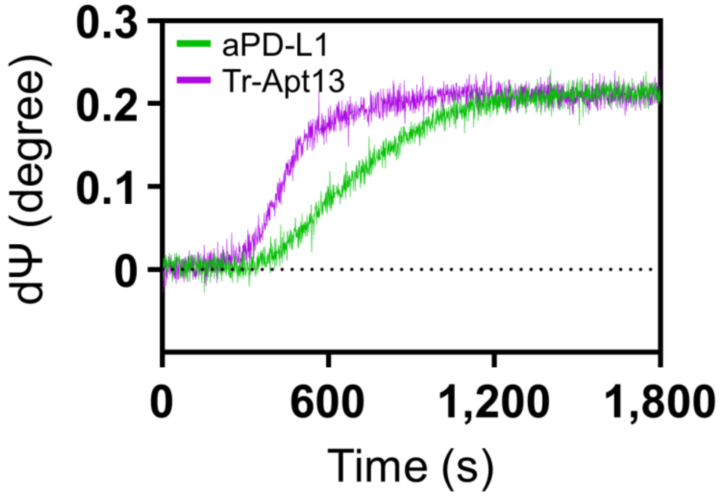

Figure S11. Ellipsometric signal measured by DP-SIS upon immobilization of Tr-Apt13 or aPD-L1 on the sensor surface. Despite its markedly lower molecular weight (10.6 kDa vs ~150 kDa), Tr-Apt13 produced a comparable increase in dΨ to aPD-L1, suggesting denser surface immobilization.

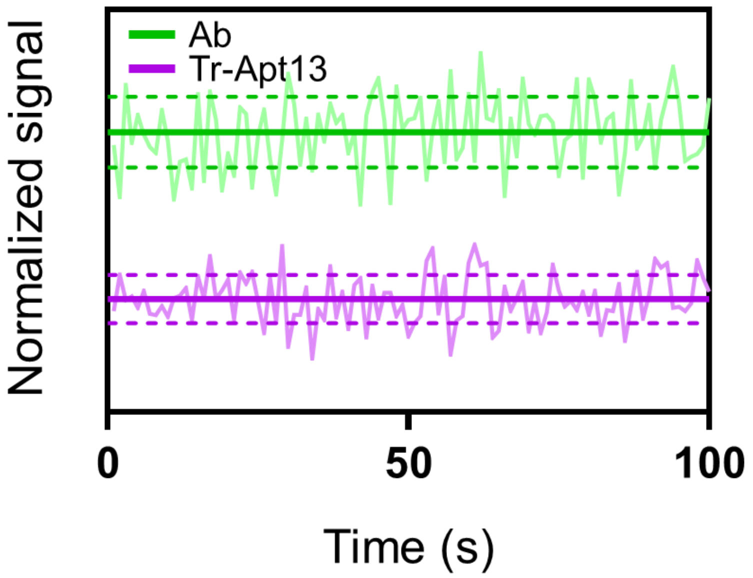

Figure S12. Signal stability analysis of DP-SIS sensor consisting of surfaces functionalized with Tr-Apt13 and aPD-L1. The Tr-Apt13–modified surface exhibited reduced fluctuations compared to the antibody-functionalized surface, demonstrating improved signal stability.

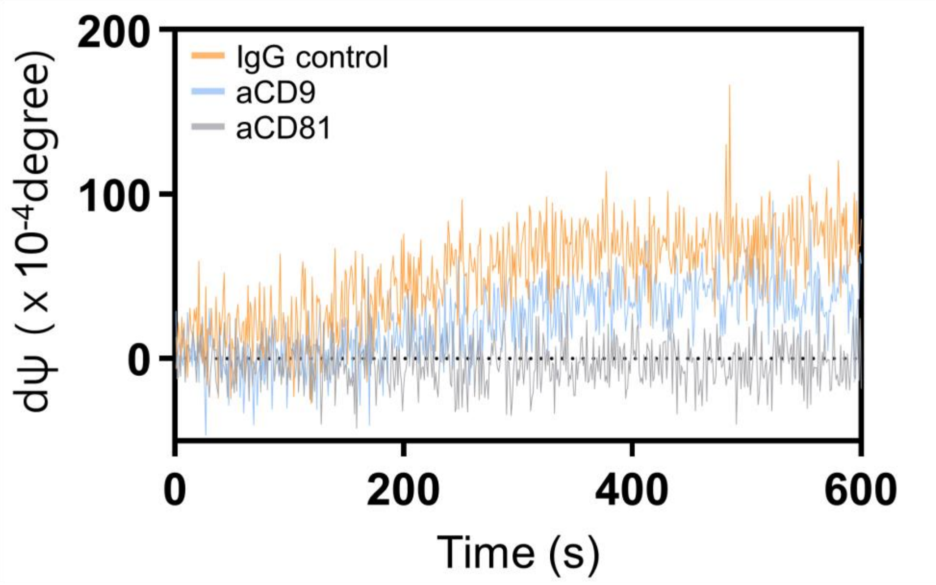

Figure S13. Post-injection of exosome-specific antibodies (aCD9 and aCD81) after capture of MDA-MB-231–derived exosomes on the Tr-Apt13–functionalized DP-SIS sensor produced an increase in dΨ, whereas IgG control showed no significant change.

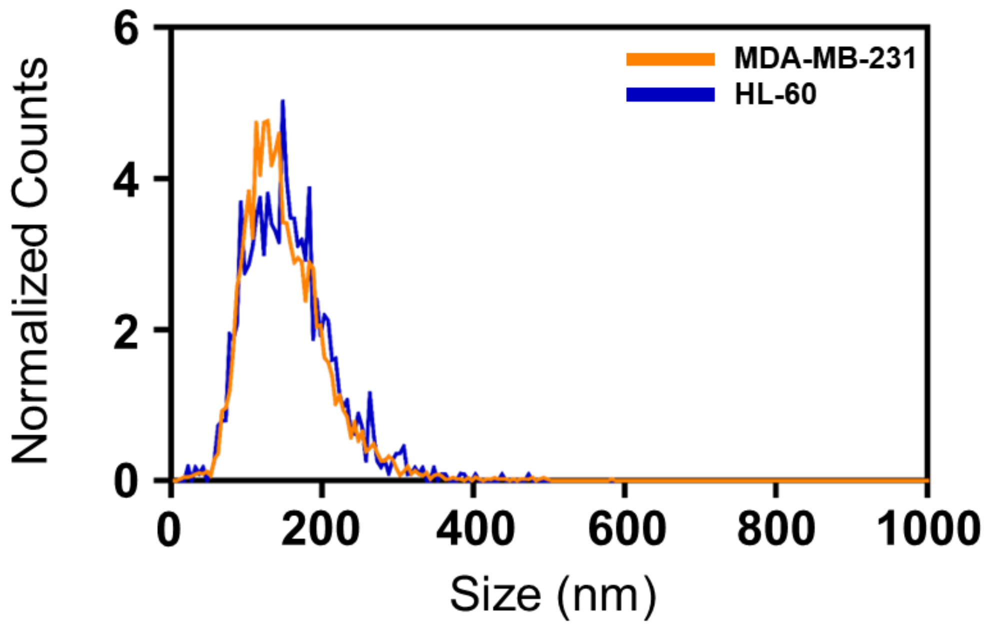

Figure S14. Nanoparticle tracking analysis of exosomes derived from PD-L1^{High} MDA-MB-231 cells and PD-L1^{Negative} HL-60 cells. Both samples exhibited a typical size distribution profile, with diameters predominantly ranging from 50 to 200 nm.

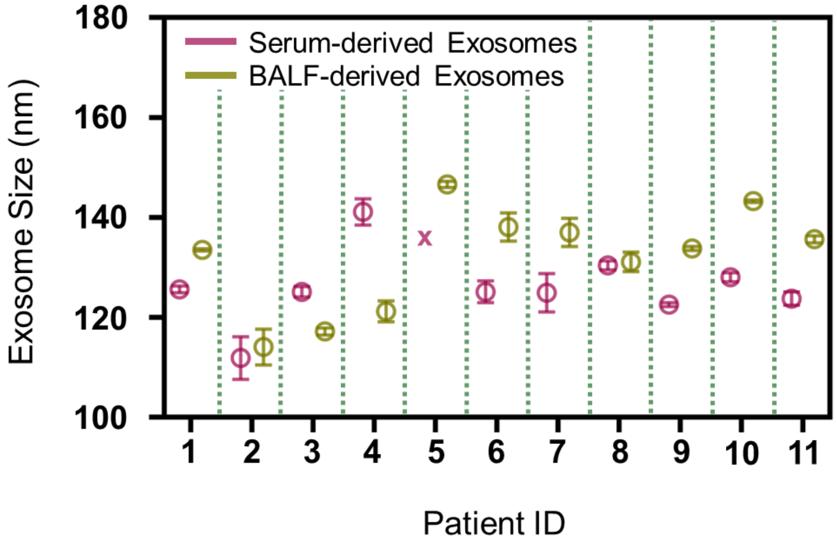

Figure S15. Nanoparticle tracking analysis of exosomes isolated from clinical BALF and serum samples. The graph shows the average particle size and standard deviation from three independent experiments, confirming no significant difference in physical size between BALF- and serum-derived exosomes. Note that for patient INHA005, only BALF-derived exosomes were analyzed.

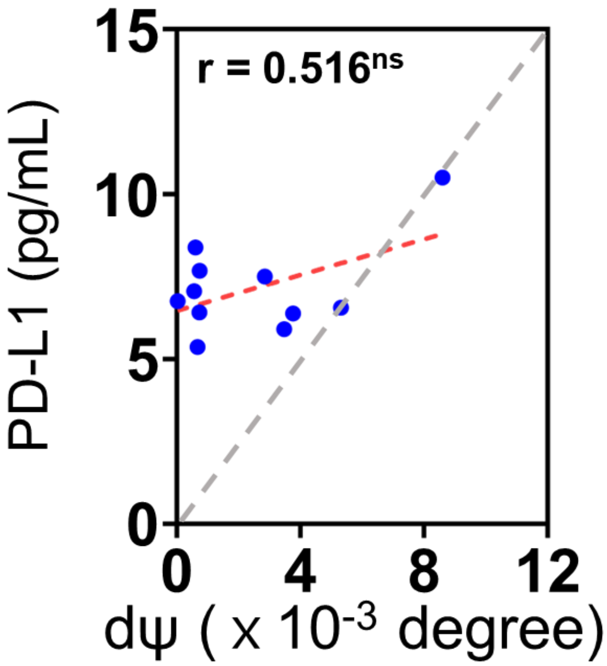

Figure S16. Pearson correlation analysis between Tr-Apt13–functionalized DP-SIS sensor signals obtained at a total BALF exosome concentration of 10 ^{3}particles/mL and exosomal PD-L1 levels quantified using ELISA (10^{6} particles/mL).

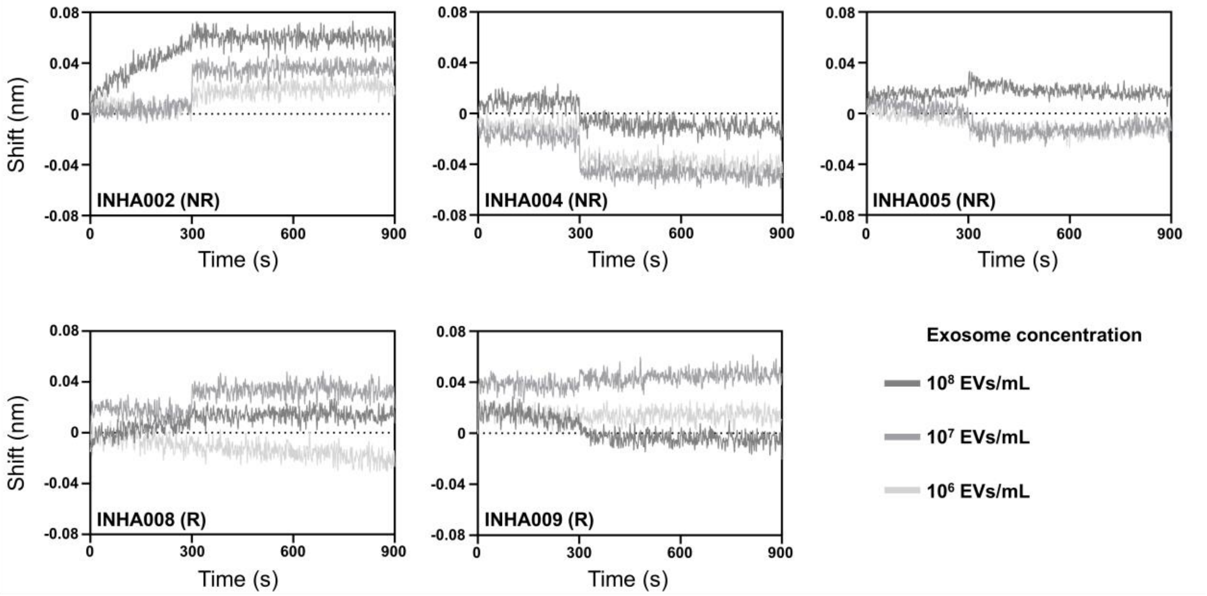

Figure S17. BLI sensorgrams of BALF-derived exosomes (10^6–10^8 particles/mL) captured on aPD-L1–immobilized chips. Only one sample (INHA002; non-responder) produced a detectable signal at 10^8 particles/mL.

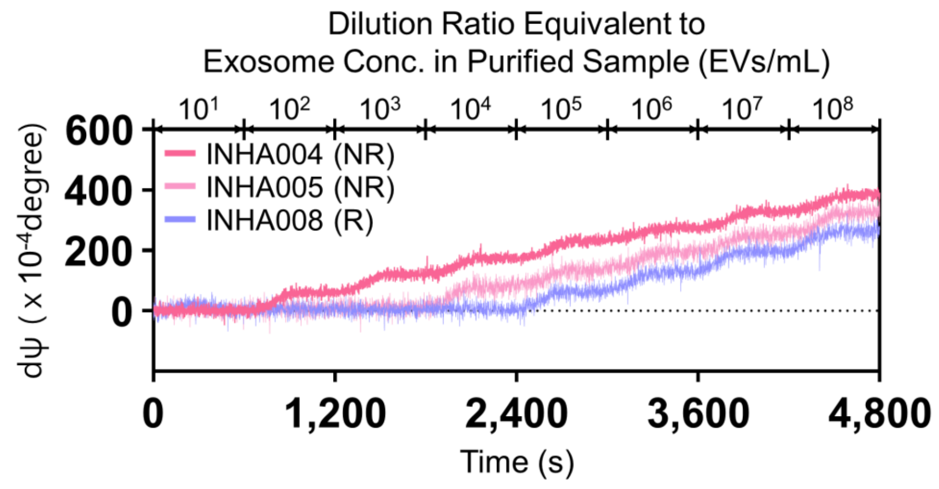

Figure S18. Direct detection of PD-L1⁺ exosomes from BALF without prior isolation using the Tr-Apt13–functionalized DP-SIS sensor. Representative sensograms from two non-responders (INHA004, INHA005) and one responder (INHA008) are shown.

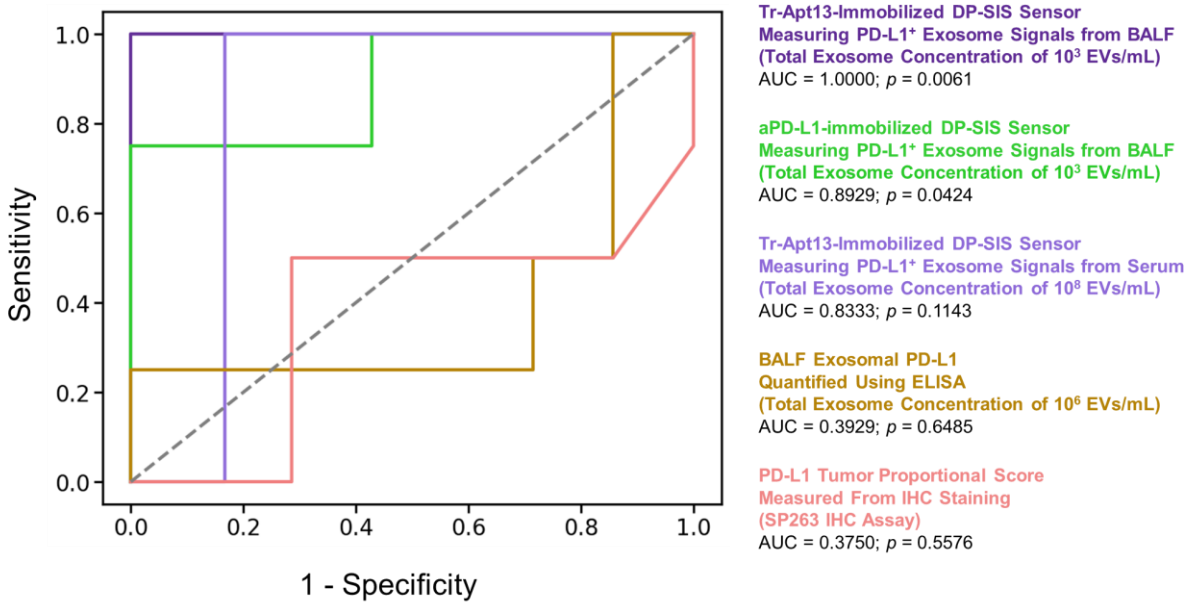

Figure S19. ROC curves comparing the predictive performance of different assays for distinguishing ICI responders from non-responders.

Table S1. PD-L1 aptamers selected by the SELEX process.

**Aptamer**	**Sequences**	**ΔG value**
PD-L1 Apt13	5’ - TGA CAC CGT ACC TGC TCT CCG CGC GAA GCA CGC CAG GGA CTA T - 3’	- 6.16 kcal/mol
PD-L1 Apt45	5’ - ATA GTC CCT GGC GTG CTT GGT CGG CAG AGC AGG TAC GGT GTC A - 3’	- 6.33 kcal/mol
PD-L1 Tr-Apt13	5’ – GT ACC T GC TCT CCG CGC GAA GCA CGC CAG GGA CT - 3’	- 5.73 kcal/mol

Table S2. Docking simulation data of Tr-APT13.

Rank	PD-L1 Tr-Apt13 docking model		
	**Docking score**	**Confidence**	**Ligand RMSD (Å)**
1	-281.52	0.9328	63.20
2	-269.41	0.9159	68.21
3	-266.36	0.9111	70.40
4	-264.92	0.9087	70.57
5	-263.02	0.9055	75.75
6	-247.24	0.8749	74.45
7	-242.07	0.8631	69.75
8	-240.48	0.8593	29.79
9	-240.04	0.8582	74.67
10	-240.02	0.8582	76.24

Table S3. Baseline clinical characteristics of the lung cancer patient cohort (*n* = 11).

Patient ID	Age	Gender	Site of Metastasis	TNM Classification				PD-L1 TPS		Best Response	Responder/ Non-responder	ICI Administered
				Stage	T	N	M	sp263	22C3			
INHA001	68	M	neck lymph node	IV	4	2	1b	50	30	PR	R	Pembrolizumab
INHA002	71	M	bone, adrenal gland	IV	3	2	1c	60	10	PD	NR	Pembrolizumab
INHA003	59	M	pleura	IV	4	2/3	0/1a	10	5	PR	R	Pembrolizumab
INHA004	57	M	bone, brain, adrenal gland, liver, contralateral lung	IV	4	3	1c	1	0	PD	NR	Pembrolizumab
INHA005	73	M	bone, brain, neck lymph node	IV	4	2	1c	70	60	PD	NR	Pembrolizumab
INHA006	76	M	bone, liver, abdominal lymph node	IV	4	3	1c	0	1	SD	NR	Pembrolizumab
INHA007	79	M	neck lymph node, abdominal lymph node	IV	3	3	1c	100	100	PR	R	Pembrolizumab
INHA008	88	M	contralateral lung	IV	2b	1	1a	5	2	PR	R	Pembrolizumab
INHA009	76	M	contralateral lung	IV	4	3	1a	15	10	PR	R	Pembrolizumab
INHA010	56	M	abdominal lymph node, contralateral lung, subcutaneous tissue	IV	4	3	1c	1	10	PR	R	Pembrolizumab
INHA011	66	M	neck lymph node, axillary lymph node	IV	2a	3	1c	100	90	PR	R	Pembrolizumab

TPS: tumor proportion score; ICI: immune checkpoint inhibitor; R: responder; NR: non-responder; PR: partial response; PD: progressive disease; SD: stable disease.

Table S4. Comparison of biomarkers and detection methods for predicting ICI responses across different cancer types.

Tumor Type (Sample Size)	ICI Treated	Biomarkers Analyzed	Detection Method	Result Summary	Ref
CRC (*n* = 180)	Tremelimumab + Durvalumab	TMB (GuardantOMNI panel)	NGS	Patients with high blood TMB showed significantly longer OS upon ICI treatment.	[58]
NSCLC (*n* = 737)	Atezolizumab Nivolumab Pembrolizumab Toripalimab Tislelizumab	Allele frequency-adjusted blood TMB (NCC-GP150, consisting of panel of 150 genes)	NGS	Low allele frequency-adjusted blood TMB better predicted OS, PFS, and response rate upon ICI treatment compared to standard blood TMB-based analysis.	[59]
Stage III Melanoma (*n* = 133)	Ipilimumab Nivolumab Pembrolizumab	ctDNA (Mutation analysis from BRAF, NRAS, RAC1, TERT, TP53, KIT)	ddPCR	Postoperative ctDNA detection was strongly predictive of relapse, while ctDNA clearance upon ICI treatment was associated with absence of recurrence.	[60]
UC (*n* = 581)	Adjuvant Atezolizumab	ctDNA (16 patient-specific clonal tumor mutations identified by WES from tissue samples.)	Multiplexed targeted PCR	Adjuvant ICI treatment improved DFS and OS only in patients with detectable postoperative ctDNA, with ctDNA clearance upon treatment correlating with favorable outcomes.	[61]
Advanced NSCLC (*n* = 39)	Nivolumab	Soluble PD-L1	ELISA	Low baseline plasma soluble PD-L1 levels were associated with better response rates and longer survival upon ICI treatment.	[62]
Metastatic NSCLC (*n* = 51)	Nivolumab	Soluble PD-1/PD-L1	ELISA	Baseline positivity for soluble PD-1 and/or PD-L1 predicted poor outcomes with nivolumab	[63]
Lung Cancer (*n* = 15)	Pembrolizumab, Atezolizumab	PD-L1-Expressing Exosomes	BCA upon capture of PD-L1-expressing exosomes	Pre-treatment PD-L1$^+$ exosome levels were associated with poor clinical outcomes and outperformed conventional PD-L1 IHC.	[64]
Melanoma (*n* = 44)	Pembrolizumab	Exosomal PD-L1 from lysates	ELISA	Changes in exosomal PD-L1 levels were associated with clinical outcome upon ICI treatment.	[65]
Lung Cancer (*n* = 11)	Pembrolizumab	PD-L1-Expressing Exosomes	Tr-Apt13-immobilized DP-SIS Sensor	PD-L1$^+$ exosomes were detected at a LOD of 9.8 × 10⁰ particles/mL, with dΨ signals significantly higher in non-responders. Elevated PD-L1$^+$ exosome levels were associated with poor PFS.	**This Study**

CRC: colorectal cancer; TMB: tumor mutation burden; NGS: next-generation sequencing; OS: overall survival; ICI: immune checkpoint inhibitor; NSCLC: non-small cell lung cancer; PFS: progression-free survival; ctDNA: circulating tumor DNA; ddPCR: droplet digital PCR; UC: urothelial carcinoma; DFS: disease-free survival; ELISA: enzyme-linked immunosirbent assay; PD-1: programmed death 1; PD-L1: programmed death-ligand 1; BCA: bicinchonicin acid assay.
